# Neuroprotective Phytochemicals in Experimental Ischemic Stroke: Mechanisms and Potential Clinical Applications

**DOI:** 10.1155/2021/6687386

**Published:** 2021-04-28

**Authors:** Hui Xu, Emily Wang, Feng Chen, Jianbo Xiao, Mingfu Wang

**Affiliations:** ^1^Institute for Advanced Study, Shenzhen University, Shenzhen 508060, China; ^2^School of Biological Sciences, The University of Hong Kong, Pokfulam Road, Hong Kong, China; ^3^Rice University, Houston, Texas, USA; ^4^International Research Center for Food Nutrition and Safety, Jiangsu University, Zhenjiang 212013, China

## Abstract

Ischemic stroke is a challenging disease with high mortality and disability rates, causing a great economic and social burden worldwide. During ischemic stroke, ionic imbalance and excitotoxicity, oxidative stress, and inflammation are developed in a relatively certain order, which then activate the cell death pathways directly or indirectly via the promotion of organelle dysfunction. Neuroprotection, a therapy that is aimed at inhibiting this damaging cascade, is therefore an important therapeutic strategy for ischemic stroke. Notably, phytochemicals showed great neuroprotective potential in preclinical research via various strategies including modulation of calcium levels and antiexcitotoxicity, antioxidation, anti-inflammation and BBB protection, mitochondrial protection and antiapoptosis, autophagy/mitophagy regulation, and regulation of neurotrophin release. In this review, we summarize the research works that report the neuroprotective activity of phytochemicals in the past 10 years and discuss the neuroprotective mechanisms and potential clinical applications of 148 phytochemicals that belong to the categories of flavonoids, stilbenoids, other phenols, terpenoids, and alkaloids. Among them, scutellarin, pinocembrin, puerarin, hydroxysafflor yellow A, salvianolic acids, rosmarinic acid, borneol, bilobalide, ginkgolides, ginsenoside Rd, and vinpocetine show great potential in clinical ischemic stroke treatment. This review will serve as a powerful reference for the screening of phytochemicals with potential clinical applications in ischemic stroke or the synthesis of new neuroprotective agents that take phytochemicals as leading compounds.

## 1. Introduction: Ischemic Stroke

Stroke occurs when the blood supply to the brain tissue is interrupted or reduced. Generally, stroke can be divided into two major categories: ischemic stroke and hemorrhagic stroke, according to how the blood flow is disrupted. Ischemic stroke is caused by the occlusion of cerebral arteries by thrombi or embolisms, blocking the blood flow to one part of the brain. Hemorrhagic stroke results from the ruptures of a weakened blood vessel, leading to the accumulation of blood in the surrounding brain tissue [1]. Of the two, ischemic stroke is the primary type, accounting for about 80% of all strokes [2]. Stroke ranks second in the cause of death worldwide, and about 5.5 million people die from stroke each year (WHO health statistics). Besides, stroke has a high disability rate, resulting in permanent disability for around 50% of its survivors [3]. Many risk factors are associated with stroke, such as age, hypertension, obesity, hyperlipidemia, diabetes, smoking, and alcohol consumption. With the great increase in the aging population, the occurrence of stroke is predicted to continue rising, and the mortality of stroke may exceed 12% by 2030 [4]. Hence, stroke is a challenging disease that greatly increases the worldwide economic and social burden.

### 1.1. Pathophysiology of Ischemic Stroke

When ischemic stroke occurs, blood flow to the specific territory of the brain that is supplied by the occluded artery is reduced. Generally, the ischemic area of the brain can be divided into the infarct core and the ischemic penumbra according to the severity of the blood flow reduction. The infarct core is characterized by a rapid decrease in ATP levels and energy stores and severe ionic disruption, which result in cell death within a few minutes. Surrounding the core area is the ischemic penumbra. In this area, blood flow reduction is less severe due to perfusion from collateral blood vessels. Hence, the insult to the ischemic penumbra is much milder than that to the infarct core. As a result, multiple milder cell death mechanisms occur in this area such as inflammation and apoptosis, providing promising therapeutic targets for ischemic stroke [5]. Notably, the ischemic penumbra is dynamic, in which the infarct core expands at the cost of the penumbra during cerebral ischemia. Hereby, early reperfusion is the most effective manner to reduce the cerebral infarction of ischemic stroke patients [6].

**Figure 1:**
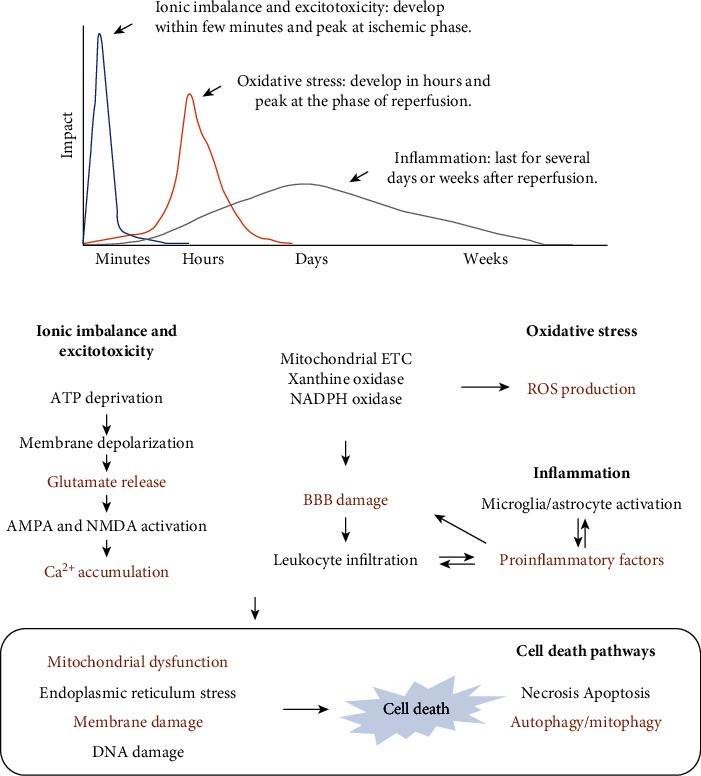
Dominant cell death mechanisms in ischemic stroke. Ionic imbalance and excitotoxicity, oxidative stress, and inflammation are major causes that lead to brain cell death in ischemic stroke. Ionic imbalance and excitotoxicity are developed within few minutes after ischemia and are the leading cause of cell death during the ischemic phase. Oxidative stress peaks at the beginning phase of reperfusion due to the sharply increased ROS production after oxygen restoration, while inflammation can last for several days or weeks after reperfusion contributing to the delayed cell death after ischemic stroke. Generally, these mechanisms can activate various cell death pathways such as necrosis, apoptosis, and autophagy/mitophagy directly or indirectly by promoting the dysfunction of organelles such as the mitochondria and endoplasmic reticulum.

Ischemic stroke injuries include two parts: ischemic injury and reperfusion injury. The cell death mechanisms of the ischemic brain are redundant, and at least three dominant mechanisms are involved: ionic imbalance and excitotoxicity, oxidative/nitrosative stress, and inflammation. Notably, those mechanisms are developed in a relatively certain order and become the dominant events at different stages of ischemic stroke. Generally, ionic imbalance and excitotoxicity play a critical role in the ischemic phase, and oxidative/nitrosative stress peaks at the beginning phase of reperfusion, while inflammation lasts for several days or weeks after reperfusion. After activation, those mechanisms affect the function of cell membranes and organelles such as the mitochondria, endoplasmic reticulum (ER), lysosomes, and nuclei. Consequently, different cell death pathways are activated, including apoptosis and necrosis [5]. Autophagy/mitophagy is also activated in ischemic stroke, but whether it promotes or decreases the cerebral ischemia-reperfusion (I/R) injuries has not been agreed upon at present. Studies suggested that apoptosis and cytoprotective autophagy/mitophagy tended to be induced by moderate cerebral I/R injuries, while necrosis or destructive autophagy/mitophagy was activated during severe I/R damage [7]. The major mechanisms of cell death in ischemic stroke are illustrated in Figure 1.

### 1.2. Major Pharmacological Therapies for Ischemic Stroke

Major approaches to treat ischemic stroke can be divided into two types: recanalization and neuroprotection. Recanalization is aimed at restoring the blood flow with thrombolytic agents or accessory devices in the acute phase of ischemic stroke (from minutes to hours) or preventing the reoccurrence of stroke with antiplatelet and anticoagulant agents, while neuroprotection is aimed at protecting neurons from the different pathological factors of ischemic stroke [8]. Recently, researchers also pronounced the theory of promoting brain neurogenesis to achieve long-term recovery after ischemic stroke. Several compounds are found to enhance neurogenesis in experimental stroke models, such as epigallocatechin-3-gallate (EGCG), curcumin, and ginkgolide K [9–11]. Yet, no agents are clinically approved for this therapy at present.

#### 1.2.1. Thrombolysis

Intravenous (IV) thrombolysis with recombination tissue plasminogen activator (r-tPA, alteplase) is the only US Food and Drug Administration- (FDA-) approved pharmacological treatment for acute ischemic stroke [12]. tPA promotes the conversion of plasminogen to plasmin, an active proteolytic enzyme that cleaves the cross-linkages between fibrin molecules of clots [13]. Notably, r-tPA has a very short therapeutic window and is best when administrated within 3 h after symptom onset. Patients can still benefit from r-tPA when it is administrated between 3 and 4.5 h after cerebral ischemia. However, r-tPA is not recommended for patients whose treatment cannot be initiated within 4.5 h because it will greatly increase the rate of intracranial hemorrhage and neuronal excitotoxicity [14]. Clinically, the short therapeutic window drastically limits the eligible patients and only about 15% of the hospitalized patients are treated with r-tPA [14].

#### 1.2.2. Antiplatelets and Anticoagulants

Antiplatelet and anticoagulant therapies are aimed at preventing the reoccurrence of stroke via the prevention of clot formation. Antiplatelets inhibit platelet activation or aggregation, while anticoagulants suppress the functions of clot-forming factors such as factors II, VII, and X. The common antiplatelet agents include aspirin, clopidogrel, dipyridamole, tirofiban, and eptifibatide. Clinical studies show that the risk of early recurrent stroke is decreased by aspirin administration within 48 h of ischemic stroke onset [15]. As for anticoagulants such as heparin, warfarin, dabigatran, rivaroxaban, and apixaban, it is found that urgent therapeutic anticoagulation benefits high-risk cardioembolic stroke patients. Yet, the use of anticoagulants may lead to symptomatic intracranial hemorrhage in unselected ischemic stroke patients [13].

#### 1.2.3. Neuroprotection

Neuroprotective agents could reduce ischemic brain injuries via the promotion of neuronal survival, neuroplasticity, synaptogenesis, and neurogenesis. Hence, neuroprotection therapy could be combined with thrombolytic agents to reduce the second injuries of reperfusion [16]. Over the past two decades, over 1000 potential neuroprotective agents were found in experimental ischemic stroke, with nearly 200 agents having undergone clinical trials [17]. Particularly, edaravone and Dl-3-n-butylphthalide show great efficacy in clinical treatment and have been approved for ischemic stroke treatment in Japan and China, respectively.

Edaravone, with the trade name Radicut/Radicava, is a medication developed by Mitsubishi Tanabe Pharma of Japan. Edaravone is a free radical scavenger that targets peroxyl radicals. It was approved for the treatment of ischemic stroke in 2001 and amyotrophic lateral sclerosis (ALS) in 2017 [18]. Edaravone is widely applied in Japan, China, and other Asian countries, and nearly half of ischemic stroke patients receive edaravone treatment in Japan [19]. Clinical studies show that the combination of edaravone and intravenous thrombolysis therapy improves the neurological outcome of ischemic stroke patients [19, 20]. Besides, edaravone is also found to reduce in-hospital mortality and intracranial hemorrhage when combined with endovascular thrombolysis therapy [21].

Dl-3-n-butylphthalide (NBP) is a neuroprotective drug developed by CSPC Pharmaceutical Group Limited. NBP is originally extracted from the seeds of *Apium graveolens*; synthesized NBP was later approved for ischemic stroke treatment in 2002. Clinically, NBP soft capsules and injections have been used to treat mild to moderate ischemic stroke patients in China. NBP is a multitargeted agent, exerting neuroprotection in ischemic stroke via antioxidation, anti-inflammation, antiapoptosis, and mitochondrial protection [22]. Clinical studies indicate that NBP improves neurological deficits such as waking, speaking, sense, thought, and memory impairments, promoting long-term recovery of ischemic stroke patients [23].

### 1.3. Common Models for Experimental Ischemic Stroke Research

#### 1.3.1. Middle Cerebral Artery Occlusion Model

Most ischemic strokes occur in the middle cerebral artery (MCA) territory of the human brain, so animal models are developed to induce ischemia in this area to mimic the clinical situation. There are several ways to occlude the MCA in experimental ischemic stroke research, and the most commonly used one is the intraluminal suture MCA occlusion (MCAO) model. In this model, a monofilament is inserted into the internal carotid artery (ICA) and advanced to the origin of MCA to block the blood flow. The monofilament can be left in the blood vessel to mimic the permanent ischemia (pMCAO) or pulled out to achieve reperfusion as a model of transient focal cerebral ischemia (tMCAO/R). Normally, 60-120 min of ischemia is commonly used in rats to induce neuronal death and cerebral infarction. In addition, MCA can also be occluded directly by clipping, ligation, or hooks through the craniectomy [2]. Robinson et al. firstly report an approach that can achieve direct occlusion of the distal MCA (dMCAO) through ligation in Sprague-Dawley rats. The dMCAO model is more reproducible than the suture MCAO model, but it may induce skull trauma, resulting in cortical inflammation and spreading depression [24, 25].

#### 1.3.2. Photothrombotic Model

The photothrombotic stroke model is induced by the intravascular photooxidation of a photosensitive dye (e.g., Rose Bengal). For stroke induction, the photosensitive dye is intravenously or intraperitoneally injected, after which the targeted cerebral vessel is illuminated with a light beam of a specific wavelength through the intact skull to activate the dye. The activated dye then promotes endothelial injuries and platelet aggregation via the formation of superoxides. Notably, the application of stereotactic coordinates during illumination makes it possible to induce infarction at the desired cortical brain region. Due to its high reproducibility and low mortality, this model is often used to study the long-term functional outcomes after stroke. Yet, the photothrombotic model has fundamental discrepancies with the pathophysiology of human ischemic stroke because of the lack of the ischemic penumbra and collateral blood flow [25–27].

#### 1.3.3. Thromboembolic Clot Model

The thromboembolic model involves the application of prepared blood clots to achieve focal cerebral vascular occlusion. The clots are usually formed spontaneously or induced by thrombin from autologous blood. Besides, injection of thrombin directly to the MCA or intracranial segment of ICA is also a common method to induce clots. This model has a high similarity to the mechanism of vascular occlusion in human ischemic stroke, so it is often used to study thrombolysis or mechanical reperfusion-related strategies [28]. For example, Ma et al. reported the effect of pinocembrin in extending the therapeutic window of r-tPA with this model [29]. However, the infarct location and size induced by the thromboembolic model are variable due to differences in the size and elasticity of clots. Hence, this model is less reproducible than the MCAO model [25].

#### 1.3.4. Global Cerebral Ischemia Model

Global cerebral ischemia is aimed at blocking all the blood flow to the brain, causing neuronal injuries to the selectively vulnerable brain areas such as the CA1 pyramidal neurons of the hippocampus and neocortex. There are many ways to achieve global cerebral ischemia including decapitation, neck tourniquet, ventricular fibrillation, and occlusion of ICAs and vertebral vessels. Currently, the most used method is bilateral ICA occlusion, namely, the two-vessel occlusion (2-VO) model. Notably, the 2-VO model induces cerebral injuries in the vulnerable brain areas with a very short ischemia period. It is found that damage can be observed in the hippocampus of animals that only suffer from 2 min of bilateral ICA occlusion. Although the global cerebral ischemia model is not fully compliant with the pathogenesis of human ischemic stroke, it still has advantages in studying the poststroke cognitive and neurological outcomes due to its selective damage to the vulnerable hippocampus [30].

## 2. Neuroprotective Strategies of Phytochemicals in Experimental Ischemic Stroke

Dominant mechanisms that lead to cell death in ischemic stroke include ionic imbalance and excitotoxicity, oxidative stress, and inflammation. After initiation, these events then activate various cell death pathways, including necrosis, apoptosis, and autophagy/mitophagy, directly or indirectly by causing the dysfunction of organelles, such as mitochondria and ER. Theoretically, all the events in this damaging cascade could be modulated to achieve potential neuroprotection in ischemic stroke. Notably, several strategies have been proved to be effective in experimental ischemic stroke, and the major strategies that are modulated by phytochemicals are reviewed in this section.

### 2.1. Calcium Modulation and Antiexcitotoxicity

Glucose and oxygen deprivation disrupts the electron transport chain (ETC), limiting the production of ATP in mitochondria. ATP depletion then enhances the anaerobic metabolism, inducing disorder of Na^+^/K^+^-ATPase and Ca^2+^/H^+^-ATPase pumps. As a result, the intracellular H^+^, Na^+^, and Ca^2+^ levels are greatly elevated, causing neuronal cell membrane depolarization and acidosis [5]. Membrane depolarization markedly elevates the release of excitatory amino acids such as glutamate. Meanwhile, the reuptake of those excitatory amino acids is impaired due to energy failure. Hence, glutamate is excessively accumulated in the extracellular space, leading to the activation of two glutamate-dependent Ca^2+^ ion channels: NMDA (N-methyl-D-aspartate) and AMPA (*α*-amino-3-hydroxy-5-methyl-4-isoxazolepropionic acid) receptors [31]. Consequently, intracellular Ca^2+^ is dramatically elevated, activating many Ca^2+^-dependent enzymes to promote necrotic and apoptotic cell death [32]. Accordingly, inhibition of intracellular Ca^2+^ accumulation or extracellular glutamate levels would reduce neuronal damage. In addition, ionic imbalance and excitotoxicity peak at the end of ischemia, so agents that target this strategy should be administrated as early as possible. Late administration could lead to ineffectiveness or even damage to brain tissues. Detailed strategies for calcium modulation and antiexcitotoxicity include enhancing the reuptake of glutamate, upregulating the inhibitory amino acid systems, modulating the activity of NMDA receptors, and regulating the non-glutamate-dependent calcium-permeable cation channels.

#### 2.1.1. Enhancing the Reuptake of Glutamate

Reuptake of glutamate is mediated by the excitatory amino acid transporters (EAATs) in astrocytes and neurons. Three types of EAATs are found in the central nervous system (CNS) of rodents, including GLAST (glutamate/aspartate transporter), GLT-1 (glutamate transporter-1), and EAAC1. Under ischemia conditions, functions of EAATs are suppressed due to ionic imbalance and ATP depletion, enhancing the neurotoxicity of glutamate. Hence, upregulation of the expression or activity of EAATs helps to reduce excitotoxicity in ischemic stroke [33]. Notably, the effectiveness of EAAT modulation has been indicated by many *in vitro* and *in vivo* studies. Several EAAT activators such as ginsenoside Rb1 and harmine have been found to possess neuroprotective activities in experimental ischemic stroke [34, 35].

#### 2.1.2. Upregulating the Inhibitory Amino Acid Systems

Inhibitory amino acids could bind to their corresponding receptors and inhibit the postsynaptic excitatory response, and the major inhibitory amino acid in the CNS is gamma-aminobutyric acid (GABA). Cerebral ischemia not only disrupts the balance between glutamate and GABA release but also suppresses the activity of GABA receptors. As a result, the inhibitory effect of GABA is markedly inhibited in ischemic stroke. Hereby, improving the glutamate and GABA balance or upregulating GABA receptors contributes to brain repair. As evidence, the GABA receptor agonist clomethiazole was reported to exert neuroprotection in animal models [36]. Besides, EGCG and ginkgolide B, two natural products that mediated neuroprotection, were found to be achieved partially by improving the balance of excitatory/inhibitory amino acids [37, 38].

#### 2.1.3. Modulating the Activity of NMDA Receptors

NMDA receptors consist of four subunits: two GluN1 and two GluN2 (glutamate-binding). Early studies regarded the NMDA receptor as a vital regulator for glutamate-mediated neurotoxicity in ischemic stroke. Hence, numerous NMDA receptor antagonists were tested to evaluate their neuroprotective activities. However, researchers found that the toxicities of NMDA receptor antagonists were high, limiting their further application. Recently, studies indicated that the high toxicity of NMDA receptor antagonists might be attributed to the dual function of NMDA receptors in ischemic stroke. It is found that the functions of NMDA receptors depend on their locations and the subunit types. Generally, the GluN2 subunit greatly affects the function of NMDA receptors. GluN2A is mainly expressed at the synapse and promotes cell survival by activating prosurvival pathways such as PI3K (phosphoinositide 3-kinase)/Akt (protein kinase B) and CREB (cyclic AMP response element-binding protein). On the contrary, GluN2B is highly expressed in extrasynaptic sites and activates prodeath pathways such as nNOS (neuronal nitric oxide synthases). During cerebral ischemia, GluN2B is the primary activated NMDA receptor, contributing to cerebral I/R injuries. Hence, selectively inhibiting GluN2B or its downstream prodeath pathways would be a neuroprotective strategy [36, 39]. For example, Tat-NR2B9c, a peptide that inhibited GluN2B-mediated prodeath pathways, was found to protect neurons in MCAO models [40]. In addition, upregulation of GluN2A was reported to contribute to neuronal survival. As evidence, geniposide enhanced the expression of GluN2A and reduced brain damage in tMCAO/R rats [41].

#### 2.1.4. Regulating the Non-Glutamate-Dependent Calcium-Permeable Cation Channels

The influx of Ca^2+^ is also modulated via non-glutamate-dependent cation channels, including TRP (transient receptor potential) channels and ASICs (acid-sensing ion channels) [42]. TRP channels can be divided into six subgroups, with TRPC6, TRPM7, and TRPV1 being extensively studied in ischemic stroke. The roles of TRPs in ischemic stroke are different. TRPM7 and TRPV1 promote neuronal death by elevating the intracellular Ca^2+^ level. Yet, TRPC6 contributes to neuronal survival via activation of CaMK (calmodulin-dependent protein kinase) and CREB signaling pathways. Notably, cerebral ischemia promotes the expression of TRPM7 and TRPV1 and enhances the degradation of TRPC6. Hereby, upregulation of TRPC6 or downregulation of TRPM7/TRPV1 would decrease cerebral I/R-induced injuries [42–44]. As an example, TRPC6 was activated by resveratrol and calycosin in MCAO models [43, 45], while inhibition of TRPM7 and TRPV1 was observed in carvacrol- and capsaicin-mediated neuroprotection, respectively [46, 47].

ASICs, especially ASIC1a and ASIC2a, are found to mediate cerebral I/R-induced injuries. Among them, ASIC1a promotes Ca^2+^ influx and neuronal injuries after being activated by the increased acidosis during ischemia, while ASIC2a reduces brain damage as observed in a transient global ischemia model [48, 49]. Modulation of ASICs was observed in ginsenoside Rd-mediated neuroprotection, which inhibited ASIC1a and enhanced ASIC2a expression in MCAO/R rats.

### 2.2. Antioxidation

Free radicals start to be produced during ischemia, surging in the reperfusion period, in which the free radical production systems such as mitochondrial ETC and enzymatic conversion systems are greatly promoted after oxygen restoration. In mitochondria, excessive Ca^2+^ accumulation during ischemia leads to the dephosphorylation of the oxidative phosphorylation (OxPhos) complexes, hyperactivating the ETC system. After reperfusion, the hyperactive ETC markedly promotes the generation of reactive oxygen species (ROS) with the supply of sufficient oxygen and glucose [50]. The enzymatic systems mainly include xanthine oxidase and NADPH (nicotinamide adenine dinucleotide phosphate) oxidase. Similarly, those enzymes are also hyperactivated during ischemia via accumulation, phosphorylation, or uncoupling, so ROS production in enzymatic systems is markedly enhanced after reperfusion [7].

Oxidative stress plays a critical role in reperfusion injuries. Firstly, oxidative stress directly destroys the cellular membrane system and DNA, leading to necrotic and apoptotic cell death. Secondly, oxidative stress enhances the opening of mPTP (mitochondrial permeability transition pore) in mitochondria, increasing the release of many proapoptotic factors such as cytochrome c and AIF (apoptosis-inducing factor). Thirdly, oxidative stress increases the permeability of the blood-brain barrier (BBB) by activating matrix metalloproteases (MMPs), thus elevating the incidence of cerebral hemorrhage, brain edema, and leukocyte infiltration [51, 52]. Finally, oxidative stress interacts with the cascade of inflammation, further deteriorating reperfusion injuries. Accordingly, antioxidation would be an important strategy to reduce cerebral I/R injuries.

Methods to modulate the oxidative stress in ischemic stroke are relatively uncomplicated, mainly including reducing NADPH oxidase-mediated ROS production and enhancing the antioxidant defense by activating the Nrf2 (nuclear factor erythroid 2-related factor 2) pathway. Yet, this strategy is found to be modulated by numerous neuroprotective agents including some phytochemicals.

#### 2.2.1. Reducing NADPH Oxidase-Mediated ROS Production

NADPH oxidase (NOX) is regarded as the primary target to modulate ROS production in ischemic stroke, in which it is relatively hard to pharmacologically inhibit the ETC. NOXs have several homologs, with NOX2 and NOX4 playing critical roles in ischemic stroke. After ischemia, NOX4 expression in neurons is markedly increased, while NOX2 upregulation is mainly found in endothelial cells. Elevated NOX promotes the generation of ROS, so inhibition of NOX would help to reduce cerebral I/R-induced oxidative stress. The effectiveness of NOX inhibition is reported in animal models. To illustrate, NOX inhibitors, such as isoquercetin, ginsenoside Rb1, picroside II, and andrographolide, were all found to protect brain tissues from cerebral I/R damage [53–57].

#### 2.2.2. Enhancing the Antioxidant Defense via the Nrf2 Pathway

Normally, ROS could be scavenged by the intracellular antioxidant defenses, including enzymatic antioxidants (e.g., SOD and catalase) and nonenzymatic antioxidants (e.g., ascorbic acid) to maintain redox homeostasis [58]. However, cerebral I/R injuries greatly promote ROS production, overburdening the antioxidant defense systems. Hereby, strengthening the antioxidant defense is a critical strategy to reduce oxidative stress in ischemic stroke. Nrf2 is the major transcriptional factor that regulates the intracellular antioxidant defense, especially under stress conditions. Once activated, Nrf2 enhances the expression of various antioxidant enzymes such as GCL (glutamate-cysteine ligase), HO-1 (heme oxygenase-1), and NQO1 (NAD(P)H dehydrogenase [quinone] 1). Overwhelming evidence indicates that Nrf2 reduces cerebral I/R-induced oxidative stress. Nrf2 activators such as sulforaphane, tert-butylhydroquinone, nobiletin, naringenin, astragaloside IV, and neferine were all reported to exert neuroprotection in experimental ischemic stroke [59–63].

### 2.3. Anti-Inflammation and BBB Protection

Inflammation is the primary poststroke damage that produces the delayed progression of cell death after ischemic stroke, developing and lasting for several days or weeks after reperfusion. Inflammation is jointly mediated by the infiltrated leukocytes and brain resident immune cells: microglia/macrophages and astrocytes. Under cerebral I/R conditions, microglia are activated rapidly and display two phenotypes: the proinflammatory phenotype (M1) and the anti-inflammatory phenotype (M2). M1 microglia contribute to neuronal cell death via secreting the proinflammatory cytokines, such as IL-1*β*, IL-6, and TNF-*α*. Yet, M2 microglia promote the recovery of the injured brain via anti-inflammatory mediators, such as IL-4, IL-10, and neurotrophins [64]. Astrocytes are activated after microglia and release multiple proinflammatory cytokines and inducible NOS (iNOS) after activation [65]. Then, the activated microglia and astrocytes promote the expression of adhesion molecules such as ICAM-1 (intercellular adhesion molecule 1) and induce the leukocyte infiltration into the ischemic brain, triggering a stronger cascade of inflammation. Worse even, the elevated level of proinflammatory factors activates MMPs and increases the BBB permeability. As a result, leukocyte infiltration is further elevated, creating a vicious cycle [66].

The inflammation cascade involves multiple regulators, so many targets could be modulated during this process. Methods to achieve anti-inflammation in experimental ischemic stroke mainly include regulation of microglial/astrocyte activation and leukocyte infiltration, inhibition of arachidonic acid release and metabolism, modulation of the transcriptional factors related to inflammation, and suppression of the TLR signaling pathway.

#### 2.3.1. Regulation of Microglial/Astrocyte Activation and Leukocyte Infiltration

As discussed above, the activated microglia/astrocytes and infiltrated leukocytes greatly promoted the release of various proinflammatory factors, such as TNF-*α*, IL-6, IL-1*β*, MCP-1 (monocyte chemoattractant protein-1), ICAM-1, and iNOS, so inhibition of microglial/astrocyte activation and leukocyte infiltration would contribute to neuroprotection. Accordingly, numerous neuroprotective agents were reported to modulate this strategy in ischemic stroke. To illustrate, scutellarin, epicatechin, fisetin, and calycosin were found to inhibit microglial activation in animal models of ischemic stroke, with epicatechin and fisetin also suppressing leukocyte infiltration [67–70]. Besides, salidroside-, ginkgolide B-, and celastrol-mediated neuroprotection were associated with the promotion of M2 microglial polarization, that is, transferring proinflammatory M1 microglia to anti-inflammatory M2 microglia [71–73]. Furthermore, berberine, harmine, and tanshinone IIA exerted neuroprotection via inhibition of astrocyte activation [35, 74, 75].

#### 2.3.2. Inhibition of Arachidonic Acid Release and Metabolism

Arachidonic acid (AA), a polyunsaturated fatty acid, is stored in the phospholipid membrane in the form of glycerol under normal conditions. Yet, elevated free radicals during ischemic stroke initiate the hydrolysis of phospholipid via activation of the phospholipases (PL, mainly PLA2 in the case of ischemic stroke). As a result, AA is released to the intracellular space and then degraded to produce several proinflammatory metabolites [76]. The degradation of AA is mediated by three independent enzymes: cyclooxygenases (COX) to form prostaglandins (PG), lipoxygenases (LOX) to form leukotrienes, and cytochrome P_450_ epoxygenases to form epoxyeicosatrienoic acids (EETs), respectively. Among them, COX-2 and 5-LOX are well studied in cerebral I/R-induced inflammation. It is shown that expressions of COX-2 and 5-LOX are increased after cerebral ischemia, and inhibition of COX-2 or 5-LOX by their corresponding inhibitors reduces brain damage in animal models. In addition, 12/15-LOX is also reported to promote cerebral ischemic injuries, as evidenced by few recent studies. Hence, many targets can be modulated in the metabolism of AA including PLA2, COX-2, 5-LOX, and 12/15-LOX [65]. For instance, apigenin-, chrysin-, and picroside II-mediated neuroprotection were related to the suppression of COX-2 [77–79]. Besides, the 5-LOX inhibitors caffeic acid and boswellic acid and 12/15-LOX inhibitors baicalein and oxymatrine were reported to reduce cerebral damage in MCAO models [80–83].

#### 2.3.3. Modulation of the Transcriptional Factors Related to Inflammation

A series of transcriptional factors participate in the cascade of inflammation, such as STAT3 (signal transducer and activator of transcription 3), NF-*κ*B (nuclear factor-*κ*B), PPAR*α* (peroxisome proliferator-activated receptor *α*), and PPAR*γ*. These transcriptional factors target diverse genes and eventually exert different functions in inflammation [84]. NF-*κ*B is a well-known proinflammatory transcriptional factor. After activation, NF-*κ*B promotes the expressions of various proinflammatory factors, such as iNOS, 5-LOX, COX-2, TNF-*α*, and IL-6. Accordingly, inhibition of the activity of NF-*κ*B is found to reduce the cerebral infarction of MCAO rodents [84]. As an example, the neuroprotective activities of nobiletin and naringenin were mediated by inhibition of NF-*κ*B [60, 85].

JAK2 (Janus kinase 2) is a receptor of proinflammatory cytokines, such as IL-6. Once activated, JAK2 promotes the phosphorylation and nuclear translocation of STAT3, initiating the expression of its target genes. The JAK2/STAT3 pathway is found to play dual roles in ischemic stroke. Some studies reported that the JAK2/STAT3 pathway contributes to brain recovery by promoting neuronal survival and neurogenesis. Yet, JAK2/STAT3 is also found to promote inflammation, especially when activated in the microglia [84]. Hence, several agents, such as atractylenolide III and sinomenine, were reported to suppress inflammation and reduce brain injuries via inhibition of the JAK2/STAT3 pathway in preclinical studies [86, 87].

PPARs are the major regulator of cellular glucose and lipid metabolism. Recent studies found that PPAR*α*/*γ* agonists exhibit anti-inflammatory activities, indicating that PPAR*α*/*γ* might also mediate the inflammatory response. PPAR*α*/*γ* are reported to suppress the inflammation cascade in ischemic stroke [84]. Accordingly, a PPAR*γ* activator, malibatol A, and a PPAR*α*/*γ* activator, icariin, were found to decrease cerebral damage in tMCAO/R models [88, 89].

#### 2.3.4. Suppression of the TLR Signaling Pathway

TLR (Toll-like receptor), a transmembrane protein, can initiate inflammation in response to exogenous or endogenous stress. TLRs have several homologs, and TLR2/4 are reported to be involved in the inflammation cascade of ischemic stroke. The activation of TLR2/4 requires endogenous ligands, such as HMGB1 (high mobility group box 1), HSPs (heat shock proteins), hyaluronic acid, and fibronectin. After combination with ligands, the configuration of TLRs is changed, leading to the recruitment of its adaptors such as MyD88 (myeloid differentiation primary response 88) and TRIF (TIR-domain-containing adapter-inducing interferon-*β*). The recruited adaptors then promote inflammation via activation of NF-*κ*B [90]. Hence, inhibition of the HMGB1/TLR/MyD88/NF-*κ*B pathway could be a potential neuroprotective strategy. Many neuroprotective compounds are reported to modulate this pathway. For instance, glycyrrhizin and berberine inhibited the HMGB1/TLR4 pathway, and vinpocetine suppressed the TLR4/MyD88/NF-*κ*B signaling [91–93]. Beyond that, baicalin-, luteolin-, and curcumin-mediated neuroprotection were also found to be associated with the inhibition of TLRs [94–96].

### 2.4. Mitochondrial Protection and Antiapoptosis

It is known that mitochondria play a vital role in reperfusion-induced injury via the generation of excessive ROS [50]. Elevated intracellular ROS and Ca^2+^ levels lead to the opening of mPTP, a complicated complex existing in the mitochondrial membrane. As a result, the permeability of mitochondria is enhanced and many mitochondrial proapoptotic factors are released such as cytochrome c and AIF [97]. Cytochrome c is a central regulator in caspase-dependent apoptosis. Released cytochrome c promotes the cascade of apoptosis via activation of caspase-9 and caspase-3. AIF is found to mainly mediate caspase-independent apoptosis. After release, AIF is translocated to the nucleus, binds to DNA, and promotes the chromatin condensation and annexin staining, initiating the apoptotic cascade [6].

Since the opening of mPTP is the major initiator for apoptosis, inhibition of the mPTP opening would be an effective neuroprotective strategy. Accordingly, hydroxysafflor yellow A-, gallic acid-, and picroside II-mediated neuroprotection were all found to be related to the inhibition of mPTP [98–100]. In addition, some regulators can modulate the opening of mPTP such as Bcl-2 family proteins and cyclophilin D. Bcl-2 proteins consist of proapoptotic proteins (e.g., Bax, Bad) and antiapoptotic proteins (e.g., Bcl-2, Bcl-xl). It is found that Bax promotes mPTP formation, while Bcl-2 could combine with Bax to inhibit its function. Hence, the ratio of Bcl-2/Bax is regarded as an important indicator of the mPTP opening, and many neuroprotective agents are found to regulate Bcl-2/Bax [97]. As an example, the ratio of Bcl-2/Bax was increased in galangin-treated pMCAO rats [101]. As for the other regulator, cyclophilin D promotes mPTP formation via binding to one of its components, the VDAC (voltage-dependent anion channel). Hereby, inhibition of cyclophilin D was also observed in the neuroprotective activities of some agents such as cyclosporin A and gallic acid [99]. The PI3K/Akt signaling pathway is a critical regulator of apoptosis. It is found that Akt promotes the phosphorylation of Bad, an inhibitor of Bcl-2. After phosphorylation, Bad separates from Bcl-2 and promotes the binding of Bcl-2 with mitochondria, suppressing the mPTP opening and subsequent cytochrome c release [102]. Since the PI3K/Akt signaling pathway is fundamental in cerebral I/R-induced apoptosis, it is modulated by most of the antiapoptotic agents in experimental ischemic stroke. For example, puerarin- and silibinin (silybin)-mediated neuroprotection were associated with the upregulation of the PI3K/Akt signaling pathway [103, 104].

### 2.5. Autophagy/Mitophagy Regulation

Autophagy is a complicated process that transports the cytoplasmic proteins or organelles to lysosomes for degradation. The process of autophagy can be divided into four main steps: initiation, prolongation, fusion, and degradation. Initiation is aimed at forming the phagophore via the ULK1-initiated cascades. Prolongation is extending and closing of the phagophore to form a matured autophagosome that contains the targeted proteins or organelles. This process is mediated by the ATG12 and LC3 ubiquitin-like conjugation systems, in which LC3 II plays central roles. The mature autophagosome is then fused with the lysosome and degraded by lysosomal enzymes [105, 106]. Autophagy is initially regarded as a nonselective process, but now it is widely accepted that autophagy can also be induced by a selective manner, such as through selective degradation of damaged mitochondria (mitophagy). The mitophagy cascade is similar to autophagy, except that it needs to detect the damaged mitochondria first. Generally, mitochondria which possess a decreased mitochondrial membrane potential (*Δψ*_m_) are identified and divided into two parts: healthy mitochondria and depolarized mitochondria. The depolarized mitochondria then initiate the mitophagy cascade and eventually are degraded. Notably, it is found that mitophagy is initiated after mitochondrial fission; that is, inhibition of mitochondrial fission will accordingly suppress the mitophagy [107].

Overwhelming evidence shows that autophagy/mitophagy is activated in various ischemic stroke models. Yet, the role of autophagy/mitophagy in cerebral I/R-induced injuries is still controversial at present. Several studies regard autophagy/mitophagy as a type of cell death, playing a detrimental role in ischemic stroke. Those studies indicate that neuronal death or brain damage is reduced after blocking the autophagy/mitophagy cascades via administration of 3-methyladenine (3-MA, an autophagosome formation inhibitor) or after knockdown of Beclin1 and Atg7, two critical regulators in the autophagy cascade in various *in vitro* and *in vivo* models [108]. Accordingly, inhibition of autophagy/mitophagy confers the neuroprotection of several agents, such as baicalein, calycosin, and puerarin [70, 109, 110].

On the contrary, autophagy/mitophagy is also found to play an important role in maintaining cellular homeostasis via degradation of defective or aggregated proteins and organelles [111]. The protective effects of autophagy/mitophagy in ischemic stroke are indicated by many investigations. For instance, Rami reported that inhibition of autophagy/mitophagy by 3-MA (3-methyladenine) or Atg7 knockdown in the reperfusion phase enhanced cytochrome c release and apoptosis both *in vitro* and *in vivo* [112]. In addition, many neuroprotective agents are reported to enhance the autophagy/mitophagy cascade in experimental ischemic stroke. To illustrate, the neuroprotective effects of triptolide, astragaloside IV, and ginsenoside Rb1 were found to be mediated by enhanced autophagy [113–115]. Besides, elevated mitophagy contributed to the neuroprotection of rapamycin, methylene blue, melatonin, and ginsenoside Rg1 in MCAO models [116, 117].

The controversial results are attributed to the differences in drug administration time points, doses, or routes [111]. Although no consensus has been reached at present, autophagy/mitophagy modulation is still considered to be a promising neuroprotective strategy due to its extensive interactions with the other cell death pathways such as necrosis and apoptosis. Yet, more studies are needed to further clarify its role in ischemic stroke.

### 2.6. Regulation of Neurotrophin Release

Neurotrophins are critical regulators of neuronal survival, development, function, and regeneration. There are many types of neurotrophins in the mammalian CNS, with NGF (nerve growth factor) and BDNF (brain-derived neurotrophic factor) being intensively studied in ischemic stroke. NGF is abundantly expressed in both the hippocampus and the cortex. After release, it binds to the TrkA (tropomyosin-related kinase A) receptor and triggers the activation of the Erk (extracellular signal-regulated kinase)/CREB pathway to improve neuronal recovery. BDNF is the most abundant neurotrophin in the mammalian CNS. BDNF binds to the TrkB receptor, activating several prosurvival pathways including PI3K/Akt signaling and MAPKs (mitogen-activated protein kinases). As one of the self-rescuing mechanisms for neurons, the expression of NGF/TrkA and BDNF/TrkB is upregulated after ischemic stroke [118, 119]. Hence, the promotion of this process would contribute to neuronal survival and recovery. For instance, rutin and astaxanthin were found to upregulate the expression of NGF or BDNF and reduced cerebral infarction and neurological deficits in MCAO models [119, 120]. In addition, some studies also evaluated the effects of exogenous neurotrophins in ischemic stroke. It is found that administration of exogenous neurotrophins exerted neuroprotection in animal models but failed to do the same in the clinical trials due to their low BBB permeability [121].

## 3. Phytochemicals That Exert Neuroprotection in Experimental Ischemic Stroke

Phytochemicals are the secondary metabolites of plants, such as vegetables, fruits, and herbs. Generally, phytochemicals can be divided into several chemical groups, including phenolics, terpenoids, and alkaloids. Phenolics are a class of compounds that possess at least one aromatic ring, with one or more hydroxyl groups attached. They can be further classified as flavonoids, stilbenes, phenolic acids, phenolic alcohols, and lignans. Terpenoids refer to the compounds that have the isoprene unit as their basic component, while alkaloids possess one or more nitrogen atoms in the heterocyclic ring [122]. Phytochemicals are famous for their antioxidative and anti-inflammatory activities, and some phytochemicals can usually act on more than one target to regulate cellular function. Chen et al. recently proposed a theory that the one-drug-multitarget strategy is more effective for ischemic stroke treatment when considering the complexity of stroke pathophysiology [123]. Hence, phytochemicals may have great potential in ischemic stroke treatment. In this section, we review the recent 10 years of research that reported the neuroprotective effects of phytochemicals in ischemic stroke. Only the phytochemicals that were tested on animal models of ischemic stroke are listed, and the ones that were studied extensively or possessed great translational potential are further discussed.

### 3.1. Flavonoids

Flavonoids include six major subgroups: flavones, flavanones, flavanols, flavonols, isoflavones, and anthocyanidins. In addition, flavonoids also largely exist in plants as glucoside derivatives, with the O-glycosidic bonds formed with different carbohydrates such as D-glucose, D-glucuronic acid, and D-galactose [124]. Totally, 46 kinds of neuroprotective flavonoids were found after searching the recent 10 years of studies in PubMed with keywords “Flavonoids, Stroke, Neuroprotection.” The neuroprotective flavonoids and their functional mechanisms are listed in Table 1, and the chemical structures of the extensively studied flavonoids are shown in Table 2.

#### 3.1.1. Flavones

Neuroprotective flavones include apigenin [77, 125], apigenin-7-O-*β*-D-(-6^″^-p-coumaroyl)-glucopyranoside (APG) [126], vitexin [127], baicalein [82, 109], baicalin [94, 128–131], chrysin [78, 132, 133], diosmin [134], ginkgetin [135], hispidulin [136], luteolin [95, 137, 138], luteoloside [139], orientin [140], nobiletin [60, 141–143], scutellarin [67, 144–147], and tricin 7-glucoside [148].

*Scutellaria baicalensis* is a traditional Chinese medicine that has long been used to treat ischemic stroke and cerebral edema [128]. Baicalein and baicalin are two principal components extracted from its roots. Baicalein was reported to improve cerebral infarction, brain edema, and neurobehavioral deficits in both the transient and permanent MCAO models [82, 109]. The neuroprotective strategies of baicalein mainly involve anti-inflammation, antiapoptosis, and antiautophagy. Cui et al. found that baicalein inhibited the 12/15-LOX/p38/cPLA2 pathway and thus reduced arachidonic acid release to inhibit inflammation [82]. Besides, baicalein also suppressed the activation of NF-*κ*B, providing another target for its anti-inflammatory activity [109]. Baicalin is also extensively studied in experimental ischemic stroke and found to improve cerebral infarction and poststroke cognitive impairments in different animal models. The most reported neuroprotective mechanism of baicalin was anti-inflammation, which was achieved via inhibition of the TLR2/4/NF-*κ*B pathway [94, 129]. In addition, baicalin-mediated neuroprotection was also related to its antioxidant effect. Xu et al. reported that baicalin possessed a marked ability to scavenge peroxynitrite and reduce peroxynitrite-induced neuronal injuries [128]. Furthermore, baicalin was observed to show neuroprotection in a diabetic MCAO/R rat model via activation of AMPK*α*- (5′ AMP-activated protein kinase *α*-) mediated mitochondrial protection [130]. Notably, baicalin had the ability to cross the BBB, reaching its peak concentration of 344 *μ*g/L in cerebrospinal fluid (CSF) after 30 min of administration (24 mg/kg, i.v.) [149]. To conclude, baicalin showed great neuroprotective efficacy and BBB permeability in experimental ischemic stroke, with great potential for clinical application [150].

Nobiletin, a polymethoxylated flavone, is mainly isolated from the peel of *Citrus* fruits. Nobiletin was found to reduce cerebral infarction, improve motor functional deficit, and enhance BBB integrity in MCAO models [60, 141–143]. Two major strategies for nobiletin-mediated neuroprotection were inhibition of the TLR4/NF-*κ*B pathway to reduce inflammation and upregulation of Nrf2/HO-1-mediated antioxidation [60, 141, 142]. In addition, nobiletin was also found to promote neuronal survival by activating cytoprotective pathways such as the BDNF/Akt/CREB pathway and the Akt/mTOR (mammalian target of rapamycin) pathway [141, 143]. Most importantly, nobiletin might be able to cross the BBB during cerebral I/R according to a study performed by Yasuda et al. They reported that nobiletin could be rapidly accumulated in the damaged region of the ischemic brain after being administrated (i.v.) with a dosage of 15 mg/kg after reperfusion onset [142].

Scutellarin (scutellarein-7-O-glucuronide) is one of the major active components of the herb *Erigeron breviscapus*. Its neuroprotection in ischemic stroke has been extensively studied with various animal models. A study found that scutellarin had stronger efficacy than edaravone for reducing the infarct volume and inflammation of pMCAO rats, implying the great potential of scutellarin in clinical application [144]. Recently, the Dengzhanxixin injection (approval number Z53021569), which uses scutellarin as one of the major components, was applied to clinical ischemic stroke treatment in China. The most studied neuroprotective mechanism of scutellarin was the suppression of microglial activation and inflammation [144, 147, 151]. Besides, scutellarin also promoted microglial-mediated astrogliosis and enhanced the expression of neurotrophins in astrocytes, implying an interglial regulation mechanism for scutellarin [67, 146]. Furthermore, scutellarin was found to improve cerebral blood flow in the ischemic brain [147]. To conclude, scutellarin is a key Chinese herbal medicine ingredient that has been primarily applied to clinical treatment, showing great potential in ischemic stroke.

#### 3.1.2. Flavanones

Flavanones including eriodictyol [152], eriodictyol-7-O-glucoside [153], hesperidin [154], naringenin [61, 85, 155], naringin [156], neohesperidin [157], and pinocembrin [29, 158–160] have been reported to be neuroprotective in ischemic stroke in the past 10 years.

Naringenin naturally exists in *Citrus* fruits, such as grapefruit and orange. Naringenin reduced cerebral infarction and poststroke neurological deficits in both the permanent and transient MCAO rats. The neuroprotective strategies of naringenin were found to inhibit NF-*κ*B to lower inflammation, reduce BBB dysfunction, and promote Nrf2-mediated antioxidation [61, 85, 155]. Naringin (naringenin-7-O-rhamnoglucoside), a glucose derivative of naringenin, is also largely present in the *Citrus* species. Naringin is famous for its strong free radical scavenging activity. Feng et al. showed that naringin improved brain damage in tMCAO/R rats by inhibiting ONOO^−^ (peroxynitrite) and its induced excessive mitophagy [156]. Yet, naringin might not have good BBB permeability. It was found that the concentration of naringin in CSF only reached the peak of 0.95 *μ*g/mL after it was administrated for 15 min (120 mg/kg, i.v.) [156].

Pinocembrin exists in propolis, honey, ginger roots, and wild marjoram. It has drawn much attention in ischemic stroke treatment in the past decade. Pinocembrin exerted neuroprotection in experimental ischemic stroke via antiapoptosis, upregulation of autophagy, and anti-inflammation [158]. Besides, it was shown that pinocembrin inhibited the activity of soluble epoxide hydrolase (sEH), an enzyme that degraded EETs (one of the AA metabolites) and lowered its induced neuronal damage [159]. Furthermore, pinocembrin was found to improve cognitive and memory impairments after it was administrated for 14 d in the global ischemia rat model [160]. Most importantly, *in vitro* studies found that pinocembrin could cross the BBB via a P-glycoprotein-conducted passive transport process [161] and extend the therapeutic time window of tPA treatment [29]. Zhao et al. reported that its neuroprotective efficacy was stronger than that of edaravone [158]. Most importantly, pinocembrin possessed high bioavailability and BBB permeability due to its good liposolubility [162]. Recently, pure synthetic pinocembrin has been subjected to a phase II clinical trial (NCT02059785) to evaluate its activity in ischemic stroke. Hereby, pinocembrin is one of the most potential drug candidates for ischemic stroke, showing great potential for clinical application.

#### 3.1.3. Flavanols

Three flavanols are found to exert neuroprotection in ischemic stroke including (−)-epicatechin (EC) [68, 163], (−)-epigallocatechin-3-gallate (EGCG) [37, 164–170], and procyanidin B2 [171].

EGCG is the most abundant catenin in green tea. EGCG has been reported to be effective in improving various CNS disorders including ischemic stroke, Alzheimer's disease, and Huntington's disease in animal models. It has been subjected to clinical trials to evaluate its efficacy in Alzheimer's disease (NCT00951834) and Huntington's disease (NCT01357681). For ischemic stroke, it was found that EGCG reduced cerebral infarction and promoted poststroke recovery in MCAO models. The neuroprotective strategies of EGCG involved the promotion of Nrf2-mediated antioxidation [166], suppression of inflammation via inhibiting microglial activation and NF-*κ*B [167, 172], antiapoptosis by activating the PI3K/Akt pathway [168], and decrease of ER stress via upregulation of TRPC6 [169]. Besides, EGCG also inhibited the basal lamina degradation of the BBB by lowering the activity of MMP-9 in tMCAO/R mice [170]. Clinical studies further indicated that EGCG decreased the level of MMP-2/9 in the plasma of ischemic stroke patients [164]. Furthermore, EGCG promoted long-term learning and memory recovery by maintaining the balance between the excitatory and inhibitory amino acids [37] and enhancing angiogenesis or neurogenesis [9, 173]. Hereby, EGCG showed neuroprotective activity via regulation of multiple targets in experimental ischemic stroke. Clinically, EGCG was found to extend the therapeutic time window of tPA [165]. Previously, EGCG was considered to possess low bioavailability and BBB permeability, which disqualify it as a drug candidate for ischemic stroke treatment. However, Wei et al. recently reported that BBB permeability was greatly enhanced in aging rats, so EGCG might have great treatment potential in aged patients [174].

#### 3.1.4. Flavonols

Neuroprotective flavonols include fisetin [69], galangin [101, 175], icariin [89, 176, 177], kaempferol-3-O-rutinoside and kaempferol-3-O-glucoside [178], kaempferide-7-O-(4^″^-O-acetylrhamnosyl)-3-O-rutinoside (A-F-B) [179], quercetin [180–182], rutin [119, 183], isoquercetin [54, 184], isorhamnetin [185], and myricetin [186].

Quercetin is widely distributed in fruits, vegetables, and grains, especially in apples and onions. Quercetin is often used as a dietary supplement due to its good antioxidant and anti-inflammatory activities. In experimental ischemic stroke studies, quercetin was found to reduce the brain injuries of pMCAO rats via multiple mechanisms, including antiapoptosis, promotion of autophagy, regulation of energy metabolism, and upregulation of PP2A (protein phosphatase 2) [180–182, 187]. Recently, quercetin was subjected to a clinical trial to evaluate its effect on improving the cerebral blood flow in the aged population (NCT01376011). However, it was found that quercetin possessed low oral bioavailability (<2%) and weak BBB permeability, limiting its pharmacological application in ischemic stroke [188]. Rutin (quercetin-3-rhamnosyl glucoside), a glycoside of quercetin, abundantly exists in buckwheat, passionflower, and apple. It was found that rutin (10 mg/kg) exerted similar neuroprotective efficacy as quercetin (50 mg/kg) in MCAO/R rats, with much lower effective doses [189]. However, a direct comparison of the efficacy of quercetin and rutin should be carried out. Rutin also inhibited the activity of MMP-9 and exerted BBB protective activity in a photothrombotic ischemic stroke model [183]. Furthermore, Liu et al. reported that rutin was a positive modulator of estrogen receptors. It enhanced the expression of estrogen receptors to upregulate the neurotrophin-mediated prosurvival pathways, such as the NGF/TrkA/CREB pathway and the BDNF/TrkB/Akt pathway in ovariectomized tMCAO/R rats [119]. Isoquercetin (quercetin-3-O-glucoside), another glycoside of quercetin, also possessed neuroprotective activity. The neuroprotective strategies of isoquercetin were found to be antioxidation mediated by Nrf2, inhibition of neuroinflammation via downregulating the TLR4/NF-*κ*B pathway, and antiapoptosis [54, 184].

#### 3.1.5. Isoflavones

In total, 6 isoflavones are found to possess neuroprotective activity in ischemic stroke, that is, calycosin [43, 70, 190, 191], calycosin-7-O-*β*-D-glucoside [192], formononetin [193], genistein [194–199], daidzein [200], and puerarin [103, 110, 201–203].

Genistein and daidzein, two major isoflavones in soybean and soy products, are described as phytoestrogens due to their structural similarity to human estrogen. Phytoestrogens can bind to the estrogen receptors and mimic the gene transcription of estrogen. However, few studies have reported the direct reaction of genistein or daidzein with estrogen receptors in ischemic stroke [204]. Instead, most of the studies focused on their antioxidant and antiapoptotic activities. It was reported that genistein or daidzein could improve neurological outcomes and reduce cerebral infarction regardless of whether it was administrated before or after MCAO via antioxidation and antiapoptosis [194, 195, 200, 205]. Besides, genistein also inhibited platelet aggregation and kept vascular reactivity in the MCAO rats, which might help to prevent clot formation [196]. Notably, genistein and daidzein played unique roles in treating postmenopausal cerebral ischemia, with several studies reporting the neuroprotective activity of genistein and daidzein in ovariectomized MCAO models. It was found that genistein pretreatment markedly decreased the neurological deficits and infarct volumes of ovariectomized MCAO rodents. The mechanisms involved the promotion of Nrf2-mediated antioxidation and inhibition of apoptosis by activating the PI3K/Akt/mTOR pathway [197–199]. Besides, equol, a metabolite of daidzein, was found to exert neuroprotection in the ovariectomized MCAO/R rats by enhancing the antioxidant defense [206]. Moreover, both genistein and daidzein could cross BBB to some extent. The efficiency of genistein was found to be below 10%, while the penetration index (AUC_CSF_/AUC_plasma_) of daidzein was about 11.96% in SD rats [207, 208].

Puerarin (daidzein-8-C-glucoside) is the major bioactive component in *Radix Puerariae* (kudzu root). Puerarin showed marked neuroprotection in experimental ischemic stroke. It was reported that puerarin reduced the brain injuries of tMCAO/R rats by suppression of autophagy, apoptosis, and inflammation [110, 201]. The anti-inflammatory activity of puerarin was achieved via upregulation of the cholinergic anti-inflammatory pathway, that is, promotion of *α*7nAchR (alpha7 nicotinic acetylcholine receptor) to inhibit the JAK2/NF-*κ*B pathway [202]. In addition, puerarin enhanced the BDNF/PI3K/Akt pathway to promote neuronal survival and poststroke recovery [103, 203]. Besides, the AUC_CSF_/AUC_plasma_ of puerarin in rats was found to be about 9.29%, implying its BBB permeability [208]. Notably, puerarin had shown the primary neuroprotective effect against ischemic stroke in clinical trials. For example, puerarin injection was subjected to a clinical trial, in which ischemic stroke patients were treated with conventional therapies plus an additional puerarin injection (400 mg/d) for one month. Results showed that puerarin injection significantly improved blood viscosity, neurological damage, and language function of ischemic stroke patients [209]. Besides, a meta-analysis of randomized controlled trials concluded that puerarin injection was effective and safe for clinical acute ischemic stroke treatment [210]. Hence, puerarin possessed great potential for clinical ischemic stroke treatment.

#### 3.1.6. Anthocyanidins, Chalcones, and Flavonolignans

Cyanidin-3-O-glucoside (anthocyanidin) [211], hydroxysafflor yellow A (chalcone) [98, 106, 212–215], xanthohumol (chalcone) [216], silybin (flavonolignan) [104, 217], and silymarin (flavonolignan) [218] are neuroprotective in experimental ischemic stroke.

Hydroxysafflor yellow A (HSYA), a chalcone, is extracted from *Carthamus tinctorius* (safflower), a Chinese medicine that is widely used in treating cerebrovascular diseases [219]. Safflower yellow for injection (approval number Z20050146) uses HSYA as the major bioactive ingredient and has been approved in China for the treatment of cerebrovascular diseases including ischemic stroke. HSYA was found to improve cerebral infarction and cognitive impairment in animal models. The neuroprotective strategies of HSYA were multiple, including antioxidation, anti-inflammation, antiapoptosis, antiexcitotoxicity, autophagy modulation, and mitochondrial protection [98, 106, 212–215, 220]. Lv et al. reported that HSYA targeted TLR4 to inhibit the NF-*κ*B activation and subsequent cascade of inflammation [212, 214]. Besides, HSYA promoted the activation of the PI3K/Akt pathway to promote cytoprotective autophagy and inhibit apoptosis in MCAO rats [106, 213]. As for mitochondrial protection, HSYA was found to improve mitochondrial function, reduce the mPTP opening, and promote mitochondrial biogenesis by inhibiting the production of phenylalanine [98, 215]. Notably, a single dose of 2 mg/kg (i.v.) HSYA was found to exert neuroprotection in tMCAO/R mice, implying its high bioavailability [212]. In addition, He reported that HSYA was detected in the brain tissue homogenate of the ischemic hemisphere in MCAO rats, with the peak concentration at 90 min after HSYA administration (i.v.), indicating the BBB permeability of HSYA [221]. Hereby, HSYA had marked neuroprotective efficacy and high bioavailability and BBB permeability, so it might be a good drug candidate for ischemic stroke.

#### 3.1.7. Summary of Flavonoids

From the current studies, baicalein and baicalin, scutellarin, pinocembrin, puerarin, and hydroxysafflor yellow A exerted great neuroprotective efficacy and high bioactivity and BBB permeability in experimental ischemic stroke. Besides, plant extracts/concentrates containing scutellarin, puerarin, and hydroxysafflor yellow A have been primarily applied in the clinical treatment of ischemic stroke due to their great neuroprotective effects. However, only pinocembrin as a single pure compound is on a phase II clinical trial at present.

### 3.2. Stilbenoids

Stilbenoids refer to a class of compounds that have two aromatic rings connected by an ethene bridge. Stilbenoids possess both the *cis* and *trans* forms, and the *trans* form is found to be more bioactive and stable [222]. In total, 7 stilbenoids are reported in the past 10 years for their neuroprotective activity in ischemic stroke, that is, resveratrol [45, 223–235], polydatin (resveratrol-3-*β*-D-glucoside) [236, 237], malibatol A (a resveratrol oligomer) [88, 238], oxyresveratrol [239], mulberroside A (oxyresveratrol-3,4′-diglycopyranoside) [240], pterostilbene [241], and 2,3,5,4′-tetrahydroxystilbene-2-O-*β*-D-glucoside [242]. Their neuroprotective mechanisms are explained in Table 3, and the chemical structures of the extensively studied stilbenoids are shown in Table 4.

Resveratrol, the most famous stilbenoid, is widely distributed in plants, such as grapes. The neuroprotective effect of resveratrol in ischemic stroke has been studied extensively, and dozens of related research articles were found. It was found that resveratrol administration, especially preconditioning, improved cerebral infarction, neurological deficits, poststroke depression, and ischemic tolerance in various animal models [223]. The neuroprotective strategies of resveratrol were multiple, including the common mechanisms such as anti-inflammation, promotion of Nrf2-mediated antioxidation, antiapoptosis, and BBB protection [224–227]. In addition, resveratrol was also reported to modulate the energy metabolism of the ischemic brain. It was found that resveratrol enhanced bioenergetic efficiency such as improving glycolysis and mitochondrial respiration efficiency to extend the window of ischemic tolerance, especially in elderly individuals [228–232]. Further studies revealed that this process was achieved by activating AMPK and SIRT1, an NAD^+^-dependent deacetylase that can induce adaptive responses under energy depletion conditions [228–232]. Furthermore, resveratrol maintained intracellular calcium homeostasis via the promotion of the TRPC6/CREB pathway and inhibition of the NMDA receptor [45] and activated sonic hedgehog signaling, a pathway that contributes to neurogenesis and neurological recovery [233, 234]. Notably, a novel study that focused on the gut-brain axis indicated that resveratrol inhibited inflammation via modulating the intestinal flora-mediated immune cell balance such as the Th1/Th2 balance and Treg/Th17 balance in the lamina propria of the small intestine, proposing an original hypothesis for resveratrol-mediated neuroprotection [235].

In summary, resveratrol possessed marked neuroprotective activity in experimental ischemic stroke and might have great potential in improving the ischemic tolerance when administrated before cerebral ischemia occurs.

### 3.3. Other Phenols

Other phenols refer to the phenolic compounds apart from flavonoids and stilbenoids, such as phenolic alcohols, phenolic acids, and lignans. In total, 20 other types of phenols are found to possess neuroprotection in ischemic stroke, including creosol [243], curcumin [10, 96, 244–249], cannabidiol [250–252], hydroxytyrosol [253], acteoside [254], hydroquinone [255], lyciumamide A [256], oleuropein [257, 258], salidroside [71, 259–264], 6-shogaol [265], 4-hydroxybenzyl alcohol [266], 4-methoxy benzyl alcohol [267], cinnamophilin [268], hyperforin [269], punicalagin [270, 271], caffeic acid [80, 272], ferulic acid [273], gallic acid [99, 274], rosmarinic acid [275, 276], and salvianolic acid A [277]. Their neuroprotective mechanisms are clarified in Table 5, and the chemical structures of the extensively studied ones are shown in Table 4.

Curcumin is extracted from the root of *Curcuma longa* (turmeric), a common spice plant in Asian countries [278]. It was found that curcumin reduced cerebral infarction, neurological deficits, and brain edema in tMCAO models regarding preconditioning or postconditioning. Curcumin is a pleiotropic agent for neuroprotection, modulating multiple mechanisms including antiapoptosis, inhibition of inflammation by downregulating TLR4, promotion of Akt/Nrf2-mediated antioxidation, suppression of autophagy by activating the PI3K/Akt/mTOR pathway, protection of mitochondria via upregulating SIRT1, the decrease of ER stress, and BBB protection [96, 244–248, 279]. In addition, curcumin promoted neurogenesis via activation of the Notch signaling pathway that improves poststroke recovery [10]. Xia et al. reported that curcumin also showed neuroprotection in a diabetic stroke model. The mechanism involved antiapoptosis and promotion of glucose uptake by activating GLUT1/3 (glucose transporter 1/3) [249]. Curcumin is one of the most popular phytochemicals in pharmacological research, with numerous clinical trials conducted for various clinical disorders including CNS diseases. As an example, curcumin has been subjected to several clinical trials for Alzheimer's disease (NCT00164749, NCT00099710, and NCT01001637). Yet, those clinical trials failed due to limited bioavailability and the BBB permeability of curcumin, limiting its clinical application in ischemic stroke. Recently, researchers were trying to enhance the bioavailability of curcumin via modulation of its chemical structure or use of the solid lipid particle method. To illustrate, Wicha et al. found that hexahydrocurcumin exerted neuroprotection with lower doses, showing better bioavailability than curcumin [280].

Cannabidiol (CBD), a phytocannabinoid from *Cannabis sativa*, is found to be nonpsychoactive and possesses cytoprotective activities. Cannabidiol has been studied in various therapeutic uses, especially for CNS disorders [281]. For ischemic stroke, cannabidiol was reported to exert neuroprotection with relatively low effective doses (single dose of 5 mg/kg, i.p.) in MCAO models [250–252]. The neuroprotective strategies of cannabidiol included antiapoptosis, antiexcitotoxicity, anti-inflammation, BBB protection, Ca^2+^ modulation, and metabolism regulation [250–252]. In addition, a meta-analysis that reviewed 34 publications of cannabidiol-mediated ischemic stroke indicated that cannabidiol markedly reduced cerebral I/R-induced infarction [282]. Most importantly, cannabidiol was highly lipophilic and could easily cross BBB, reaching a relatively high concentration quickly after administration [283]. Notably, more than 100 clinical trials related to the therapeutic application of cannabidiol are being conducted or are completed at present, with several trials for CNS disorders. For example, two phase II clinical trials are being conducted to evaluate the efficacy of cannabidiol on motor and tremor symptom improvement in Parkinson's disease (NCT03582137, NCT02818777).

Salidroside is the main bioactive component of *Rhodiola rosea* L. [284] and showed neuroprotection for ischemic stroke in various MCAO models. The major neuroprotective strategies of salidroside involved anti-inflammation and antiapoptosis. Liu et al. found that salidroside suppressed inflammation by promoting microglial M2 polarization [71]. Other studies showed that salidroside inhibited NF-*κ*B and activated HIF*α* (hypoxia-inducible factors) via upregulation of the PI3K/Akt pathway, providing another mechanism for its anti-inflammatory activity [261, 263, 264]. In addition, Zhang et al. reported that the antiapoptotic effect of salidroside was achieved via activation of the BDNF/PI3K/Akt pathway in tMCAO/R mice [262]. Furthermore, salidroside upregulated the cytoprotective transcriptional factor Egrs (early growth response genes) to improve neuronal activity and synaptic plasticity [259, 260]. However, the concentration of salidroside in the brain is extremely low after administration (15 mg/kg, i.v.), indicating that salidroside might have difficulty crossing BBB [285].

Salvianolic acids are the bioactive compounds extracted from the roots of *Salvia metrorrhagia* (Danshen), a traditional Chinese medicine for treating cardiovascular disease [286]. Salvianolic acid A was reported to improve brain damage in MCAO/R rats by its antioxidant, anti-inflammatory, and metabolism regulatory activities [277]. Salvianolic acids for injection (SAFI), a commercially available Chinese herb medicine developed by Tianjin Tably Pride Pharmaceutical Company, has been approved for the treatment of ischemic stroke in the recovery phase in China. SAFI is composed of five natural phenolic acids: salvianolic acid B (68.31%), salvianolic acid D (3.7%), salvianolic acid Y (5.1%), alkannic acid (3.86%), and rosmarinic acid (2.68%) [287]. In experimental ischemic stroke, SAFI was found to promote poststroke recovery through two major mechanisms: promotion of neurogenesis via activation of the sonic hedgehog pathway and via upregulation of neurotrophins such as BDNF and NGF [288]. Besides, SAFI also reduced brain damage in the acute phase of ischemic stroke. The mechanisms involved anti-inflammation via inhibition of microglial activation, as well as maintaining mitochondrial permeability in astrocytes through activation of the PI3K/Akt/mtCx43 (mitochondrial connexin 43) pathway [286, 287].

Caffeic acid is widely present in dietary plants such as fruits, vegetables, coffee, and olive oils. It was found that caffeic acid reduced cerebral infarction and improved poststroke learning, memory, and spatial deficits in global ischemia or pMCAO models [80, 272]. The mechanisms involved suppression of oxidative stress, inhibition of 5-LOX-induced inflammation, and reduction of synaptic dysfunction by upregulation of synaptophysin, a biomarker for synaptic density and plasticity [80, 272]. Rosmarinic acid, an ester of caffeic acid, exists in plants of the *Lamiaceae* family such as rosemary and perilla. Rosmarinic acid attenuated brain damage and memory deficits by promoting synaptogenic activity, suppressing inflammation, and upregulating BDNF in pMCAO mice. In addition, rosmarinic acid also exerted neuroprotection in a diabetic ischemic stroke model by promoting BBB function and inhibiting inflammation. As mentioned above, rosmarinic acid is one of the components of SAFI, accounting for 2.68% of the injection.

In summary, salvianolic acids and rosmarinic acid have been primarily applied as key ingredients in a Chinese herbal medicine for clinical ischemic stroke treatment. Yet, there is no clinical data to prove their individual effectiveness at present. Cannabidiol showed good neuroprotective efficacy and high BBB permeability, so it was a good candidate for ischemic stroke drug development. Although curcumin and salidroside showed high neuroprotective efficacy, the poor bioavailability and BBB permeability might limit their further clinical applications.

### 3.4. Terpenoids

Terpenoids are a class of compounds that have an isoprene unit as their basic component. Depending on the number of isoprene units, terpenoids can be divided into monoterpenoids (two units), sesquiterpenoids (three units), diterpenoids (four units), triterpenoids (six units), and tetraterpenoids (eight units). In total, 56 terpenoids were found to possess neuroprotective activity for ischemic stroke after searching the recent 10 years of studies in PubMed with keywords “Terpenoids, Stroke, Neuroprotection.” The neuroprotective terpenoids and their functional mechanisms are listed in Table 6, and the chemical structures of the extensively studied ones are shown in Table 7.

#### 3.4.1. Monoterpenoids

The neuroprotective monoterpenoids include borneol [289, 290], carvacrol [46, 291–293], catalpol [294, 295], cornin [296], genipin [297], geniposide [41], linalool [298], *β*-myrcene [299], paeoniflorin [300–302], perillaldehyde [303], perillyl alcohol [304], *α*-pinene [305], picroside II [56, 79, 100, 306, 307], and safranal [308].

Borneol is present in the plants of *Artemisia* and *Dipterocarpaceae* and has been used in traditional Chinese medicine to restore consciousness after stroke, coma, or other brain injuries for more than 1500 years [309]. There are three types of borneols naturally existing in the herbs: (−)-borneol, (+)-borneol, and isoborneol. Dong et al. compared the neuroprotective effect of these three borneols in pMCAO rats, finding that (−)-borneol possessed the strongest protective efficacy in reducing cerebral infarction, neurological deficits, and brain edema. The mechanism of (−)-borneol included antiapoptosis, anti-inflammation, and protection of the neurovascular units [289]. Notably, the most attractive property of borneol was its strong BBB permeability. Hereby, borneol was usually used as an “assistant” drug in traditional Chinese medicine to deliver neuroprotective medications into the brain, enhancing their therapeutic efficacy [310]. Notably, (+)-borneol possessed synergistic effects with edaravone. It was found that (+)-borneol enhanced the neuroprotective efficacy of edaravone, promoted edaravone-mediated long-term recovery, and extended its therapeutic window in the MCAO model [290]. Accordingly, a sublingual tablet consisting of edaravone and borneol was subjected to a phase I clinical trial to test its safety, tolerability, and pharmacokinetics (NCT03495206). Recently, the edaravone and dexborneol concentrated solution for injection (approval number H20200007), developed by Simcere Pharmacological Company, was approved in China in 2020. Hence, borneol has great potential to be used as an upper ushering drug in ischemic stroke.

Carvacrol is one of the major ingredients in the essential oil of oregano and thyme and is widely used as a food additive. Studies showed that it was lipophilic and able to cross BBB [311]. Carvacrol was found to reduce cerebral infarction and neurological deficits in MCAO models [46, 291–293]. Neuroprotective strategies of carvacrol involved anti-inflammation via inhibition of NF-*κ*B and antiapoptosis via suppression of TRPM7 and promotion of the PI3K/Akt pathway [46, 292]. Besides, carvacrol also improved poststroke learning and memory recovery in the global ischemia gerbil model via antioxidation and inhibition of ferroptosis, a programmed cell death pathway induced by iron ions and ROS [291]. Notably, carvacrol possessed an extended therapeutic window, exerting protective effects even when administrated (i.c.v.) at 6 h after reperfusion [293].

#### 3.4.2. Sesquiterpenoids

In total, 8 sesquiterpenoids are found to exhibit neuroprotection in ischemic stroke, including alantolactone [312], atractylenolide III [86], bakkenolide IIIa [313], bilobalide [314–317], (−)-*α*-bisabolol [318], parthenolide [319], patchouli alcohol [320], and *β*-caryophyllene [321].

The *Ginkgo biloba* leaf extract EGb761, consisting of flavonol glycosides (24%) and terpene lactones (6%), has been reported to exhibit neuroprotection in different CNS disorders such as stroke and Alzheimer's disease [322]. Bilobalide, one of the major bioactive terpenoids in EGb761 (accounting for 3% of EGb761), is widely studied in ischemic stroke. It was found that bilobalide showed neuroprotection in tMCAO/R models regardless of whether it was administrated before or after ischemia [314–317]. The neuroprotective mechanisms of bilobalide are multiple. It was found that bilobalide restored the energy supply via protection of complex I in mitochondria and reduced the damage induced by energy depletion such as glutamate release, intracellular Ca^2+^ accumulation, and mitochondrial swelling [315, 316]. Besides, bilobalide also possessed antioxidant and anti-inflammatory activities by upregulation of JNK1/2 and p38 [314, 323]. Furthermore, bilobalide promoted angiogenesis and inhibited apoptosis and autophagy via activation of the Akt/eNOS pathway in MCAO/R rats [317]. As for the BBB permeability, it was found that a significant level of bilobalide could be detected in the rat brain after administration of a single dose (8 mg/kg, i.v.), indicating the brain uptake of bilobalide [324].

#### 3.4.3. Diterpenoids

Neuroprotective diterpenoids include andrographolide [57, 325–328], erinacine A [329], ginkgolide B [38, 72, 330, 331], ginkgolide K [11, 332], pseudopterosin A [333], salvinorin A [334], tanshinone I [335], tanshinone IIA [75, 336–343], totarol [344], triptolide [113, 345, 346], and (1S,2E,4R,6R,-7E,11E)-2,7,11-cembratriene-4,6-diol [347].

Andrographolide is the primary bioactive compound in *Andrographis paniculata*, a traditional Chinese medicine that possesses anti-inflammatory, antiviral, and antibacterial activities [348]. Pharmacodynamic studies have shown that andrographolide could cross the BBB, so it was extensively studied in various CNS disorders such as ischemic stroke, Alzheimer's disease, and multiple sclerosis [349]. Recently, andrographolide was subjected to a phase II clinical trial (NCT02280876) to evaluate its efficacy in multiple sclerosis. For ischemic stroke, andrographolide was reported to reduce cerebral infarction and neurological deficits in both the transient and permanent MCAO models. The neuroprotective strategies of andrographolide mainly involved anti-inflammation via inhibition of NF-*κ*B and HIF-1*α*, antioxidation via upregulation of Nrf2/HO-1 and suppression of NOX2, and BBB protection [57, 325–327]. Besides, andrographolide also promoted long-term cognitive recovery in a global ischemia model by enhancing the BDNF/TrkB pathway [328]. Notably, andrographolide was able to cross BBB and possessed relatively low toxicity and high bioavailability, as only a single dose of 10 *μ*g/kg (i.v.) was needed to exert neuroprotective activity against tMCAO/R injuries in rats [327]. Hereby, andrographolide might be a great candidate for ischemic stroke treatment.

Ginkgolides, another group of major terpenoids in EGb761, are diterpenoids and have been applied to clinical ischemic stroke treatment in China for a decade. Two intravenous injections that contain ginkgolides as active ingredients are approved as Chinese herbal medicine to treat mild to moderate cerebral infarction in China: ginkgolide injection and ginkgolide meglumine injection. Ginkgolide injection (approval number Z20110035), containing bilobalide, ginkgolide A, ginkgolide B, and ginkgolide C, has been manufactured by Chengdu Baiyu Pharmaceutical Co., Ltd., since 2012. Ginkgolide meglumine injection (GMI, approval number Z20120024), produced by Jiangsu Kanion Pharmaceutical Co., Ltd., excludes bilobalide and only contains ginkgolide A (1.6 mg/mL), ginkgolide B (2.9 mg/mL), and ginkgolide K (0.19 mg/mL) as active ingredients [350]. Recently, several studies reported the neuroprotective mechanism of GMI in animal models. To illustrate, Geng et al. reported that GMI improved energy metabolism, reduced oxidative stress, and maintained cerebral homeostasis using a metabolomic profiling method [350]. Another study revealed that GMI targeted the PI3K/Akt pathway to activate cytoprotective transcriptional factors such as Nrf2 and CREB [351]. Notably, the neuroprotective efficacy of GMI was found to be as strong as edaravone [351]. Apart from the injections, some pure ginkgolides were also extensively studied in ischemic stroke. For example, ginkgolide B was found to be a natural platelet-activating factor (PAF) receptor antagonist and inhibited the microglial activation via inhibition of the PAF receptor in tMCAO/R mice [72]. Besides, ginkgolide B also possessed multiple neuroprotective strategies including anti-inflammation via suppression of NF-*κ*B, antiapoptosis, antiexcitotoxicity, and BBB protection [38, 330, 331]. In addition, ginkgolide K was also reported to exert neuroprotection in MCAO models. The neuroprotective mechanisms of ginkgolide K involved antioxidation, mitochondrial protection, elevation of autophagy via upregulation of the AMPK/mTOR/ULK1 signaling pathway, and promotion of neurogenesis by activating JAK2/STAT3 [11, 332, 352, 353]. A study compared the neuroprotective efficacy of ginkgolides (A, B, and K) and bilobalide and indicated that ginkgolide B exerted the strongest activities to reduce cerebral infarction and oxidative stress via the promotion of the Akt/Nrf2 pathway [354]. To conclude, ginkgolides and bilobalide have been primarily applied to clinical treatment, showing great potential in ischemic stroke.

Tanshinones are the major components in the root or rhizome of *Salvia miltiorrhiza* (Danshen), a Chinese medicine that is traditionally used to treat cardiovascular diseases. More than 40 tanshinones were found in the Danshen extract, with tanshinone I and tanshinone IIA being widely studied in the ischemic stroke area [355]. Tanshinone I was found to reduce neuronal death via anti-inflammation in global cerebral ischemic gerbils [335]. Tanshinone IIA was found to improve cerebral infarction and poststroke recovery regardless of administration before or after ischemia. The major mechanisms for tanshinone IIA-mediated neuroprotection were anti-inflammation and antiapoptosis. Anti-inflammatory activity of tanshinone IIA was achieved via suppression of the proinflammatory cytokines HMGB1 and MIF, which then downregulated the MAPKs, upregulated the PPAR*γ*, and inhibited the astrocyte activation [75, 337, 339, 340, 342, 343]. Tanshinone IIA-mediated antiapoptosis was reported to be regulated by activation of the PI3K/Akt pathway [336, 341]. In addition, tanshinone IIA improved neuronal survival and synaptic plasticity by promotion of the BDNF/CREB pathway and elevation of TORC1 (transducers of regulated CREB), a CREB coactivator [338]. Hereby, tanshinone IIA showed marked neuroprotective activity in experimental ischemic stroke. However, tanshinone IIA possessed poor solubility and half-life, limiting its BBB permeability. Accordingly, various methods were developed to enhance its bioavailability. As an example, Liu et al. developed a drug delivery system for tanshinone IIA, called cationic bovine serum albumin-conjugated tanshinone IIA PEGylated nanoparticles (CBSA-PEG-NPs). They indicated that CBSA-PEG-NPs increased the brain delivery efficiency of tanshinone IIA and thus enhanced its neuroprotective activity in ischemic stroke [339, 340].

Triptolide, a major bioactive diterpenoid in *Tripterygium wilfordii*, is famous for its anti-inflammatory and immunosuppressive activities. Studies showed that triptolide possessed good neuroprotective efficacy with very low effective doses (single dose of 0.2 mg/kg, i.p.) in tMCAO/R rats [345]. The major neuroprotective strategy of triptolide was anti-inflammation via inhibition of the p38/NF-*κ*B pathway [345, 346]. Besides, triptolide also lowered BBB permeability, suppressed apoptosis, and enhanced autophagy in the MCAO rats [113, 346]. Yet, triptolide exhibited high toxicity on the liver and the heart, limiting its clinical application [356].

#### 3.4.4. Triterpenoids

Triterpenoids are the most popular group of terpenoids and the major constituents of decoction and the extracts of many medical plants. Triterpenoid saponins, the glycosides of triterpenoids, are an important form of the bioactive triterpenoids. In total, 20 triterpenoids are found to exhibit neuroprotection in ischemic stroke including arjunolic acid [357], asiatic acid [358, 359], boswellic acids [81, 360, 361], 28-O-caffeoyl betulin [362], celastrol [73, 363], echinocystic acid [364], 18*β*-glycyrrhetinic acid [365], maslinic acid [366], ursolic acid [367], and triterpenoid glycosides: madecassoside [368], astragaloside IV [62, 114, 369, 370], glycyrrhizin [91, 371–373], diammonium glycyrrhizinate [374], ginsenoside Rb1 [34, 55, 115, 375, 376], ginsenoside Rd [49, 377–383], ginsenoside Rg1 [384–389], ginsenoside Rg3 [390], pseudoginsenoside F11 [391, 392], and notoginsenoside R1 [393].

Boswellic acids are present in the gum resin of the herb *Boswellia serrata*. Several types of boswellic acids are found in *B*. *serrata*, and two of them, namely, acetyl-11-keto-*β*-boswellic acid (AKBA) and 11-keto-*β*-boswellic acid (KBA), were reported to exert neuroprotection in ischemic stroke [394]. The major neuroprotective strategies of AKBA involved anti-inflammation via inhibition of 5-LOX and promotion of Nrf2/HO-1-mediated antioxidation [81, 360]. Similar to AKBA, the activation of the Nrf2/HO-1 pathway was also observed in KBA-mediated neuroprotection [361]. Yet, boswellic acids were reported to have poor solubility and half-time, largely restricting their pharmacological applications. Efforts were made to develop high-efficiency delivery systems for boswellic acids. For example, Ding et al. reported an AKBA-loaded O-carboxymethyl chitosan nanoparticle system and found that this system enhanced the neuroprotective efficacy of AKBA [81].

Celastrol is another major bioactive terpenoid in the root of *Tripterygium wilfordii*, in addition to the diterpenoid triptolide. Celastrol exerted neuroprotection in ischemic stroke mainly via its anti-inflammatory activity. It is reported that celastrol promoted the polarization of microglia/macrophages from the proinflammatory M1 phase to the anti-inflammatory M2 phase by decreasing proinflammatory cytokine IL-33 and its corresponding receptor ST2 (growth stimulation expressed gene 2) in pMCAO rats [73]. Besides, celastrol also inhibited the JNK/NF-*κ*B pathway to suppress the inflammatory cascade in the ischemic brain [363]. The neuroprotective efficacy of celastrol was relatively high, improving cerebral infarction and neurological deficits with low effective doses (1-3 mg/kg, i.p.). Yet, similar to triptolide, celastrol also had high toxicity and poor solubility problems, which limited its pharmacological applications [356].

Astragaloside IV is a saponin abundant in the dry root of *Astragalus membranaceus* (Huangqi), a Chinese herbal medicine that has been used to treat ischemic stroke in China for a long time [395]. Notably, astragaloside IV is usually regarded as a quality control marker of Huangqi. A meta-analysis study revealed that astragaloside IV reduced cerebral infarction, improved neurological impairments, decreased brain edema, and enhanced BBB integrity in experimental ischemic stroke [395]. The neuroprotective mechanisms of astragaloside IV were multiple including antioxidation, antiapoptosis, anti-inflammation, autophagy modulation, and mitochondrial protection [62, 114, 369, 370]. Hexokinase-II (HK-II), an enzyme for glycolysis, inhibited the mPTP opening after binding to mitochondria. Astragaloside IV was found to enhance the activity of HK-II and promote its binding to mitochondria, thus inhibiting mitochondrial dysfunction and mitochondrial apoptosis, as well as improving glycolysis to alleviate energy depletion [369]. Besides, astragaloside IV also activated the Nrf2 pathway to reduce oxidative stress and BBB permeability in an LPS-injured mouse model [62].

Ginseng, the roots of *Panax ginseng*, has been widely used in East Asian countries as a medication for thousands of years. Studies showed that ginsenosides are the major bioactive ingredients that contribute to the numerous therapeutic effects of ginseng. There are more than 180 kinds of ginsenosides extracted from ginseng. Generally, they can be divided into two groups: the protopanaxadiol group, including Rb1, Rb2, Rd, and Rg3, and the protopanaxatriol group, including Rg1, Rf, and Re. Among them, ginsenosides Rb1, Rd, Rg1, and Rg3 were reported to exert neuroprotective activities in ischemic stroke [396].

Ginsenoside Rb1 (GS-Rb1) is the most abundant ginsenoside in ginseng, accounting for about 31% of all ginsenosides in Chinese/Korean ginseng. Wang et al. found that GS-Rb1 improved the abnormal microenvironment of the hippocampus in the photothrombotic cerebral ischemia model, as evidenced by the reduced excitotoxicity, intracellular Ca^2+^ level, and apoptosis, and improved regional cerebral blood flow. The mechanisms for those improvements are reported to be upregulation of GLT-1 and inhibition of NMDA receptors and cytochrome c release [34]. Besides, GS-Rb1 inhibited the NOX4-mediated ROS production and thus reduced BBB permeability due to downregulated MMP-9 activation [55]. Furthermore, GS-Rb1 was found to enhance cytoprotective autophagy, neurogenesis, and BDNF release in MCAO models [115, 376]. Notably, Dong et al. showed that GS-Rb1 also exerted neuroprotective activity in aged mice [375].

Ginsenoside Rd (GS-Rd) has been regarded as one of the important markers for the quality of ginseng. GS-Rd showed marked neuroprotective activity in animal models regardless of preconditioning or postconditioning. Besides, GS-Rd has a relatively wide therapeutic window. Ye et al. found that GS-Rd improved cerebral infarction and neurological outcomes even when administrated after 4 h of ischemia in MCAO rats [383]. The neuroprotective mechanisms of GS-Rd were multiple including anti-inflammation, antioxidation, and antimitochondrial apoptosis via inhibition of PARP-1 (poly(ADP-ribose) polymerase 1) [377, 379–382]. Besides, GS-Rd also inhibited DNA damage via upregulation of NEIL1/3 (human endonuclease VIII-like proteins) and reduced intracellular Ca^2+^ accumulation by suppression of TRPM7 and ASIC [49, 378]. Most importantly, GS-Rd could effectively cross the intact BBB and was reported to have much stronger neuroprotective efficacy than edaravone [383]. Hereby, GS-Rd might possess great potential for clinical ischemic stroke treatment. Accordingly, GS-Rd was subjected to clinical trials, including a phase II trial (NCT00591084) and a phase III trial (NCT00815763). In total, 190 ischemic stroke patients in phase II and 390 patients in phase III were recruited. They were intravenously injected with GS-Rd (10, 20 mg) within 72 h after ischemic stroke onset for 14 d [382]. Clinical results showed that GS-Rd improved the NIHSS (National Institutes of Health Stroke Scale) at 15 d, with no significantly elevated mortality or adverse effects [397]. Hence, GS-Rd is one of the most potential drug candidates for ischemic stroke treatment.

Ginsenoside Rg1 (GS-Rg1) accounts for about 23% of all ginseng-derived ginsenosides in Chinese/Korean ginseng. It was indicated that GS-Rg1 possessed equivalent neuroprotective efficacy to GS-Rb1 in tMCAO/R rats [398]. The neuroprotective strategies of GS-Rg1 included anti-inflammation, antiexcitotoxicity, antioxidation via inhibition of miR-144, promotion of angiogenesis via activation of the PI3K/Akt/mTOR pathway, and upregulation of BDNF [384–386, 388]. In addition, GS-Rg1 also protected BBB integrity and reduced brain edema by inhibiting aquaporin-4, a water channel protein highly expressed in the astrocyte foot [387, 389]. Yet, the bioavailability, BBB permeability, and half-time of GS-Rg1 were poor, limiting its clinical application [399].

#### 3.4.5. Tetraterpenoids

In total, 3 tetraterpenoids are reported to be neuroprotective in ischemic stroke, including astaxanthin [120, 400, 401], fucoxanthin [402], and lutein [403]. Notably, they all belong to the xanthophyll type of carotenoids.

Astaxanthin, a well-known antioxidant, exists abundantly in algal species, such as *Haematococcus pluvialis*, and crustaceans. As the only carotenoid that could cross the BBB according to the present studies, astaxanthin has received much attention in ischemic stroke research [404]. It was found that pretreatment with astaxanthin decreased cerebral infarction and neurological deficits in tMCAO/R rats via antioxidation and antiapoptosis [120, 400, 401]. Besides, astaxanthin also promoted neurogenesis and the release of neurotrophins such as BDNF and NGF [120, 401]. Notably, a clinical trial (NCT03945526) was conducted to test the effect of astaxanthin supplementation (2 × 8 mg for 7 d) on plasma MDA levels and neurological deficits of ischemic stroke patients.

#### 3.4.6. Summary of Terpenoids

From the present studies, borneol, bilobalide, ginkgolides, and ginsenoside Rd have been preliminarily applied to clinical ischemic stroke treatment, and the effectiveness of ginsenoside Rd was further indicated by the clinical data. Besides, carvacrol, andrographolide, and astaxanthin were also great candidates for ischemic stroke treatment due to their high bioavailability and BBB permeability in rodent models. In addition, triptolide and celastrol showed marked neuroprotective efficacy and high bioavailability in experimental ischemic stroke. Yet, they possessed high toxicity, limiting their further clinical application. Generally, terpenoids exhibit strong neuroprotective activity in experimental ischemic stroke. However, the solubility and BBB permeability of terpenoids such as tanshinones, boswellic acids, and celastrol are poor. Although several strategies, such as the development of the nanoparticle delivery systems, have been tried to solve this limitation, no effective strategies have been officially approved at present.

### 3.5. Alkaloids

Alkaloids refer to a class of natural compounds that have one or more nitrogen atoms in the heterocyclic ring. Alkaloids can be produced in many species of plants, especially flowering plants, in the form of organic acids, esters, or binding with sugars and tannins rather than free bases. Totally, 19 natural alkaloids were found to exert neuroprotection after searching the research of the past 10 years, including berberine [74, 92, 405–409], boldine [410], capsaicin [47, 411], dihydrocapsaicin [44, 412–414], harmine [35], higenamine [415], neferine [63], nicotine [416], levo-tetrahydropalmatine [417], oxymatrine [83, 418], oxysophoridine [419], piperine [420], rhynchophylline [421], sinomenine [87, 422, 423], solasodine [424], sophoridine [425, 426], tetrandrine [427], trigonelline [428], and vinpocetine [93, 429]. Their neuroprotective mechanisms are explained in Table 8, and the chemical structures of the extensively studied alkaloids are shown in Table 7.

Berberine is an isoquinoline alkaloid present in the Chinese medicine *Rhizoma coptidis* (Huanglian). It was found that berberine could cross the BBB and accumulate in the brain tissue, so it has been extensively studied in CNS disorders including ischemic stroke [409]. Berberine exerted neuroprotection in both the global and transient cerebral ischemia models via two major mechanisms: antiapoptosis and anti-inflammation. The antiapoptosis strategy was mainly achieved by activating the PI3K/Akt pathway [74, 405, 407]. Yang et al. found that berberine enhanced the expression of BDNF and its receptor TrkB to promote the activation of the PI3K/Akt pathway [407]. Other studies further indicated that berberine could promote the activity of the PI3K p55*γ* subunit and enhance Akt-mediated GSK activation to suppress neuronal apoptosis [405, 408]. As for the anti-inflammatory strategy, it was found that berberine reduced microglial and astrocyte activation and enhanced AMPK-dependent microglial M2 polarization [406, 409]. Besides, the HMGB1/TLR4 pathway was also involved in inhibiting the activation of NF-*κ*B and the subsequent inflammatory cascade [92, 408]. In addition, Zhu et al. also reported the role of berberine in promoting angiogenesis in tMCAO/R mice [409]. Hereby, berberine showed marked neuroprotection in experimental ischemic stroke and possessed great potential for clinical application in ischemic stroke.

Capsaicin and dihydrocapsaicin are the main capsaicinoids that contribute to the pungency of chili peppers. Capsaicin and dihydrocapsaicin are famous TRPV1 antagonists, possessing desensitizing effects on TRPV1 [47]. Hence, capsaicin- and dihydrocapsaicin-mediated neuroprotection were mainly attributed to the inhibition of TRPV1. To illustrate, capsaicin was found to reduce neuronal and neurovascular damage via inhibition of TRPV1-induced excitotoxicity [47, 411]. Besides, inhibition of TRPV1 also led to hypothermia, a state that has been proved to have neuroprotective effects in experimental ischemic stroke [413]. Hereby, dihydrocapsaicin has been shown to possess marked neuroprotection via pharmacological induction of hypothermia in MCAO/R models [44, 413]. It was reported that dihydrocapsaicin-induced hypothermia protected the ischemic brain through multiple mechanisms, including antiapoptosis via activation of the PI3K/Akt pathway and promotion of Nrf2-mediated antioxidation and anti-inflammation [412–414]. Yet, it was indicated that the bioavailability and half-time of capsaicin and dihydrocapsaicin were low, limiting their clinical application. Hence, researchers are searching for the proper delivery system for capsaicin and dihydrocapsaicin.

Sinomenine is the major bioactive ingredient in the herb *Sinomenium acutum*. Sinomenine is known for its immunosuppressive activity and has been used to treat rheumatoid arthritis in China [87]. For neuroprotection, sinomenine was found to reduce cerebral infarction, neurological deficits, and brain edema in tMCAO/R rodents via its anti-inflammatory activity [87, 422, 423]. Qiu et al. reported that sinomenine upregulated AMPK to inhibit the activation of the NLRP3 (NOD-like receptor pyrin 3) inflammasome, an activator for the release of proinflammatory cytokines [422]. Besides, it was shown that sinomenine also promoted the activation of DRD2 (astrocytic dopamine D2 receptor), an anti-inflammatory factor in astrocytes, and the expression and nuclear translocation of CRYAB (*α*B-crystallin), a heat shock protein that is regulated by DRD2 [87]. Furthermore, sinomenine inhibited cerebral I/R-induced acidosis and intracellular Ca^2+^ accumulation in tMCAO/R rats by suppressing ASIC1a and L-type calcium channels [423, 430]. Notably, sinomenine was able to cross the BBB as indicated by Wu et al. that 0.11 *μ*g/g was accumulated in the rat brain after 0.5 h administration of sinomenine (10 mg/kg, i.p.) [430].

Vinpocetine is naturally present in the periwinkle plant and has been investigated at length for its effect against ischemic stroke. The neuroprotective strategy of vinpocetine in ischemic stroke mainly involved anti-inflammation via inhibition of the TLR4/MyD88 pathway [93, 429]. Notably, vinpocetine also showed a neuroprotective effect in a phase II clinical trial (NCT02878772), in which 60 ischemic stroke patients were divided into two groups: half of the patients only received standard treatment, while the other half of the patients received 30 mg/d (i.v. for 14 d) vinpocetine treatment plus standard treatment [431]. Results showed that vinpocetine reduced the secondary infarction enlargement and NF-*κ*B-mediated inflammation and improved poststroke neurological functional recovery [431]. More importantly, vinpocetine had high BBB permeability, greatly enhancing its bioavailability in CNS disorders [431]. Accordingly, vinpocetine might be one of the most promising candidates for ischemic stroke treatment.

In summary, vinpocetine and berberine had high BBB permeability and showed great neuroprotective efficacy in experimental ischemic stroke. Besides, vinpocetine also showed neuroprotective activity in the phase II clinical trial. Hence, vinpocetine and berberine might possess great potential in clinical ischemic stroke treatment. As for capsaicin and dihydrocapsaicin, although they showed good neuroprotective efficacy in experimental ischemic stroke, their poor bioavailability and BBB permeability might limit their further clinical applications.

## 4. Conclusion

Phytochemicals have been well studied in experimental ischemic stroke due to their marked neuroprotective activities. In this review, we listed 148 phytochemicals that were reported to exhibit neuroprotection in various animal models of ischemic stroke, including flavonoids (46), stilbenoids (7), other phenols (20), terpenoids (56), and alkaloids (19). Notably, several phytochemicals have been primarily applied in clinical ischemic stroke treatment or have shown neuroprotective activities in clinical trials. Those phytochemicals include scutellarin, pinocembrin, puerarin, hydroxysafflor yellow A, salvianolic acids, rosmarinic acid, borneol, bilobalide, ginkgolides, ginsenoside Rd, and vinpocetine. However, the clinical application was mainly carried out in China with purified/concentrated plant extracts or a mixture of several compounds. In addition, many phytochemicals, such as baicalein and baicalin, CBD, carvacrol, andrographolide, astaxanthin, and berberine, showed great neuroprotective efficacy and high BBB permeability and bioavailability in experimental ischemic stroke research. Hence, they also possessed great potential for clinical application. However, other agents such as naringenin, curcumin, EGCG, capsaicin, dihydrocapsaicin, and tanshinone IIA exhibited marked neuroprotective efficacy in experimental ischemic stroke but had poor solubility and BBB permeability. For those phytochemicals, modification of their chemical structures or development of efficient drug delivery systems is needed to enhance their BBB permeability.

## Figures and Tables

**Table 1:** Neuroprotective flavonoids and their functional mechanisms and targets^a^.

Compounds	Mechanisms and targets	Ref.
Flavones (15)		
Apigenin	Anti-inflammation: iNOS↓, COX-2↓, p-p38↓, p-JNK↓; histone deacetylases↓; BDNF/CREB/Syn-1↑	[77, 125]
APG	Antioxidation: p-STAT3↑	[126]
Vitexin	Antiapoptosis: p-Erk↑, p-JNK↓, p-p38↓	[127]
Baicalein	Anti-inflammation: NF-*κ*B↓, p-MAPKs↓, arachidonic acid release↓: 12/15-LOX/p38 MAPK/cPLA2↓; antiapoptosis; antiautophagy: PI3K/Akt/mTOR↑	[82, 109]
Baicalin	Antiapoptosis: p-CaMKII↓; antioxidation: peroxynitrite scavenging↑; anti-inflammation: TLR2/4/NF-*κ*B↓; mitochondrial function↑: Drp-1↓, Mfn2↓, AMPK*α*1↑	[94, 128–131]
Chrysin	Anti-inflammation: NF-*κ*B↓, COX-2↓, iNOS↓; antioxidation	[78, 132, 133]
Diosmin	Bcl-2/Bax↑; JAK2/STAT3↑	[134]
Ginkgetin	Antioxidation; anti-inflammation: JAK2/STAT3/SIRT1↓	[135]
Hispidulin	NLRP3-mediated pyroptosis↓; AMPK/GSK3*β*↑	[136]
Luteolin	Anti-inflammation: TLR4/5/p38/NF-*κ*B↓; antioxidation; antiapoptosis	[95, 137, 138]
Luteoloside	Anti-inflammation: PPAR*γ*↑/Nrf2↑/NF-*κ*B↓	[139]
Orientin	Antioxidation; anti-inflammation: TLR4/NF-*κ*B/TNF-*α*↓; AQP-4↓	[140]
Nobiletin	Anti-inflammation: TLR4/NF-*κ*B↓; antioxidation: Nrf2/HO-1↑; antiapoptosis: Akt/mTOR↑; BDNF-Akt/CREB↑; BBB permeability↓	[60, 141–143]
Scutellarin	Anti-inflammation: ACE/Ang II/AT1R↓, microglial activation↓, microglial-mediated astrogliosis↑, Notch-1/Nestin↑; neurotrophin expression↑: BDNF/NGF/GDNF-Akt/CREB↑; antioxidation	[67, 144–147]
Tricin 7-glucoside	Anti-inflammation: NF-*κ*B activation↓, HMGB1 expression↓	[148]
Flavanones (7)		
Eriodictyol	Anti-inflammation	[152]
Eriodictyol-7-O-glucoside	Antioxidation in astrocytes: Nrf2/ARE↑	[153]
Hesperidin	Antioxidation: NO pathway↓	[154]
Naringenin	BBB protection: NOD2/RIP2/NF-*κ*B/MMP-9↓; antiapoptosis; anti-inflammation: NF-*κ*B↓; antioxidation: Nrf2	[61, 85, 155]
Naringin	ONOO^−^-mediated excessive mitophagy↓	[156]
Neohesperidin	Antiapoptosis; antioxidation: Akt/Nrf2/HO-1↑	[157]
Pinocembrin	Antiapoptosis; autophagy↑; anti-inflammation: sEH/EETs↓; neuronal loss↓; astrocyte proliferation↓	[29, 158–160]
Flavanols (3)		
(−)-Epicatechin (EC)	Anti-inflammation: microglial activation↓; antioxidation: Nrf2/HO-1↑	[68, 163]
(−)-Epigallocatechin-3-Gallate (EGCG)	Calcium modulation and antiexcitotoxicity: TRPC6 degradation↓/MEK/Erk/CREB↑, balance between the excitatory and inhibitory amino acids↑; antiapoptosis: PI3K/Akt/eNOS↑; antioxidation: Nrf2/ARE↑; anti-inflammation: NF-*κ*B↓; BBB protection: MMP-2↓, MMP-9↓; ER stress↓	[37, 164–170]
Procyanidin B2	BBB protection; antioxidation: Nrf2↑	[171]
Flavonols (10)		
Fisetin	Anti-inflammation: macrophage infiltration↓, microglial activation↓, JNK/NF-*κ*B↓	[69]
Galangin	Microenvironment of the neurovascular unit (NVU)↑: Wnt/*β*-catenin↑, HIF-1*α*/VEGF↑; mitochondrial protection and antiapoptosis: Bax/Bcl-2↓	[101, 175]
Icariin	HDAC↓/CREB↑; SIRT1/PGC-1*α*↑; anti-inflammation: PPAR*α*/*γ*↑, NF-*κ*B↓	[89, 176, 177]
Kaempferol-3-O-rutinoside (KRS)/glucoside (KGS)	Anti-inflammation: STAT3↓, NF-*κ*B↓	[178]
Kaempferide-7-O-(4^″^-O-acetylrhamnosyl)-3-O-rutinoside	Anti-inflammation; antioxidation; antiapoptosis	[179]
Quercetin	Energy metabolism↑; antioxidation; PP2A subunit B↑; antiapoptosis	[180–182]
Rutin	Estrogen receptors↑: BDNF/TrkB/Akt↑ and NGF/TrkA/CREB↑; BBB protection: MMP-9 activity↓	[119, 183]
Isoquercetin	Antiapoptosis; anti-inflammation and antioxidation: Nrf2↑, NOX4/ROS/NF-*κ*B↓, MAPK/TLR4/NF-*κ*B↓	[54, 184]
Isorhamnetin	Nrf2/HO-1↑; iNOS/NO↓	[185]
Myricetin	Anti-inflammation: p38/NF-*κ*B↓; antioxidation; p-Akt↑	[186]
Isoflavones (6)		
Calycosin	Anti-inflammation: microglial activation↓; antiapoptosis; antiautophagy; BDNF/TrkB↑; calcium modulation: TRPC6/CREB	[43, 70, 190, 191]
Calycosin-7-O-*β*-D-glucoside	BBB protection: NO↓/Cav-1↑/MMPs↓	[192]
Formononetin	Bax/Bcl-2↓; PI3K/Akt↑	[193]
Genistein	Antioxidation: Nrf2↑; antiapoptosis: PI3K/Akt/mTOR↑; Erk activation↑; ROS/NF-*κ*B↓; antiplatelet aggregation; vascular protection	[194–199]
Daidzein	ROS production↓	[200]
Puerarin	Antiautophagy; anti-inflammation: neutrophil activation↓, HIF-1*α*↓, *α*7nAchR↑; antiapoptosis: PI3K/Akt1/GSK-3*β*/MCL-1↑; BDNF secret↑	[70, 109, 110]
Anthocyanidins, chalcones, and flavonolignans (5)		
Cyanidin-3-O-glucoside	Antiapoptosis: oxidative stress-induced AIF release↓	[211]
Hydroxysafflor yellow A (HSYA)	Antioxidation↓; anti-inflammation: TLR4/MAPK/NF-*κ*B↓; antiapoptosis: PI3K/Akt/GSK3*β*↑, mPTP opening↓; neurotrophin release↑: BDNF↑, GFAP↑, NGF↑; autophagy↑: Akt↑; mitochondrial function and biogenesis↑; phenylalanine synthesis↓	[98, 106, 212–215]
Xanthohumol	Anti-inflammation; antiapoptosis; platelet activation↓	[216]
Silibinin/silybin	Anti-inflammation; antioxidation; antiapoptosis and antiautophagy: PI3K/Akt/mTOR↑	[104, 217]
Silymarin	Antioxidation; antiapoptosis	[218]

^a^Notes: ↑: activation or upregulation; ↓: inhibition or downregulation. Abbreviations do not appear in the text. ACE: angiotensin-converting enzyme; Ang II: angiotensin II; AT1R: angiotensin type 1 receptor; AQP-4: aquaporin-4; Drp-1: dynamin-related protein 1; GFAP: glial fibrillary acidic protein; Mfn2: mitofusin 2; NOD2: nucleotide oligomerization domain 2; RIP2: receptor-interacting protein kinase 2; Syn-1: synaptophysin-1; VEGF: vascular endothelial growth factor.

**Table 2:** Chemical structures of some representative neuroprotective flavonoids.

Flavones			
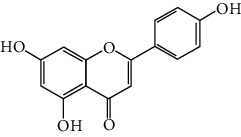	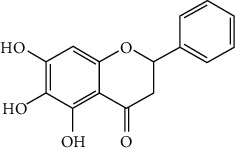	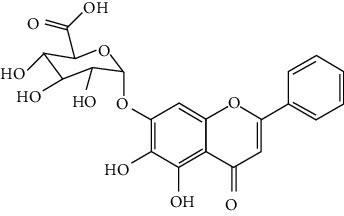	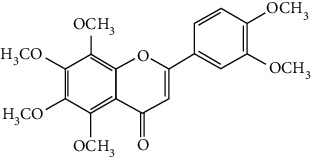
Apigenin	Baicalein	Baicalin	Nobiletin
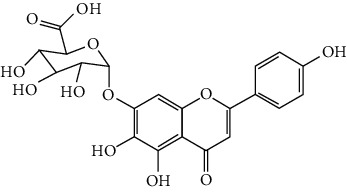	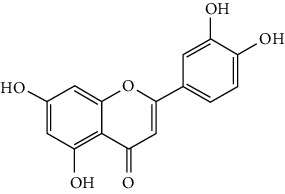	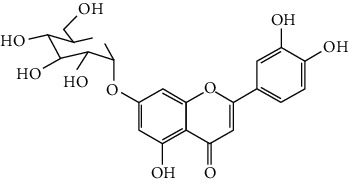	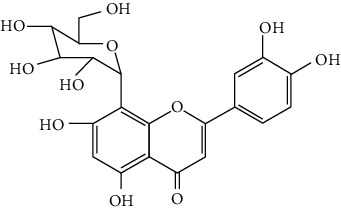
Scutellarin	Luteolin	Luteoloside	Orientin
Flavanones			
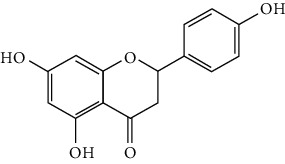	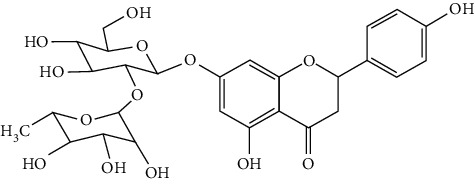		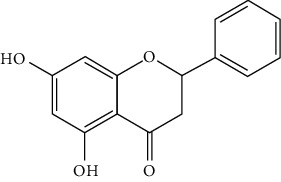
Naringenin	Naringin		Pinocembrin
Flavanols and chalcones			
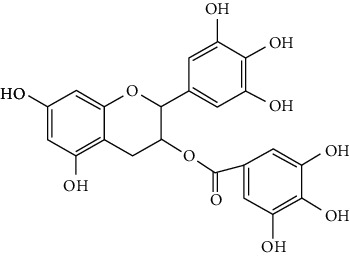		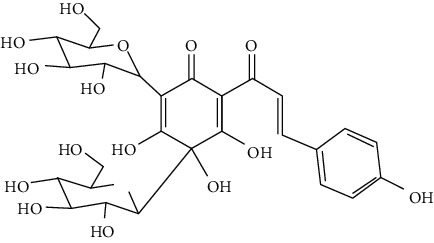	
(−)-Epigallocatechin-3-gallate (EGCG)		Hydroxysafflor yellow A (HSYA)	
Flavonols			
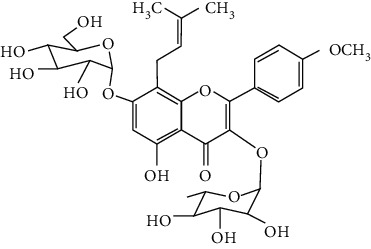	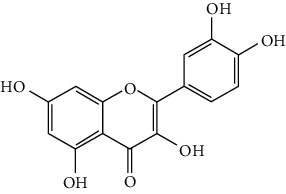	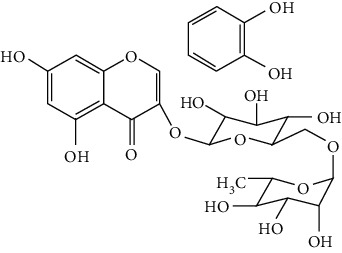	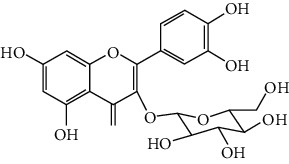
Icariin	Quercetin	Rutin	Isoquercetin
Isoflavones			
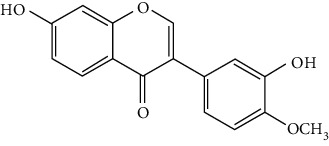	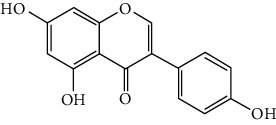	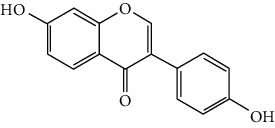	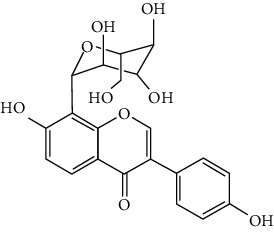
Calycosin	Genistein	Daidzein	Puerarin

**Table 3:** Neuroprotective stilbenoids and their functional mechanisms and targets^b^.

Compounds	Mechanisms and targets	Ref.
Stilbenoids (7)		
Resveratrol	Anti-inflammation: T regulatory cells (Treg)↑, intestinal flora-mediated immune cell balance↑; calcium modulation: TRPC6/MEK/CREB↑, TRPC6/CaMKIV/CREB↑, NMDA receptor↓; BDNF↑; modulating energy metabolism and extending the cerebral ischemic tolerance: glycolysis↑, mitochondrial respiration efficiency↑, phosphodiesterase↓, cAMP/AMPK/SIRT1↑, UCP2↓; antioxidation: Nrf2/HO-1; antiapoptosis; synaptic transmission efficiency↑; BBB protection: MMP-9/TIMP-1 balance↑; regulation of hypothalamus-pituitary-adrenal axis function; hedgehog signaling pathway↑; estrogen receptor↑; cellular stress proteins↑	[45, 223–235]
Polydatin	BBB protection; sonic hedgehog pathway↑; anti-inflammation: NF-*κ*B↓; antioxidation; antiapoptosis	[236, 237]
Malibatol A	Mitochondrial dysfunction↓; anti-inflammation: microglial M2 polarization↑, PPAR*γ*↑	[88, 238]
Oxyresveratrol	Antiapoptosis	[239]
Mulberroside A	Anti-inflammation: MAPK/NF-*κ*B↓	[240]
Pterostilbene	Antioxidation; antiapoptosis	[241]
2,3,5,4′-Tetrahydroxystilbene-2-O-*β*-D-glucoside	Angiogenesis↑	[242]

^b^Notes: ↑: activation or upregulation; ↓: inhibition or downregulation. Abbreviation does not appear in the text. UCP2: uncoupling protein 2.

**Table 4:** Chemical structures of some representative neuroprotective nonflavonoid phenols.

Stilbenoids			
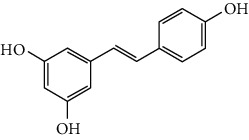	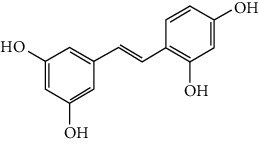	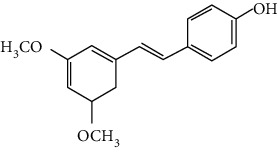	
Resveratrol	Oxyresveratrol	Pterostilbene	
Other phenols			
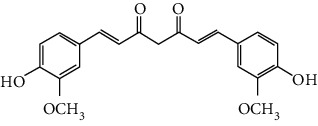	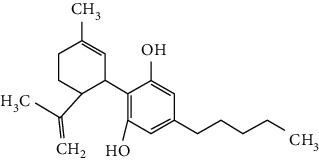	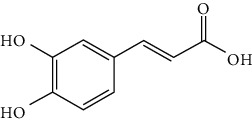	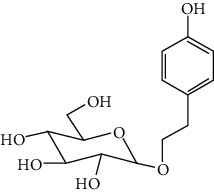
Curcumin (keto form)	Cannabidiol	Caffeic acid	Salidroside
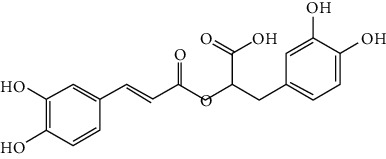	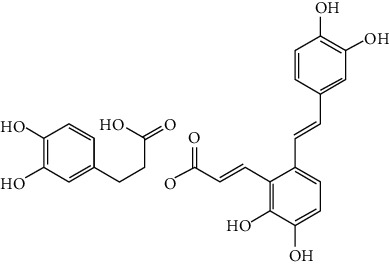	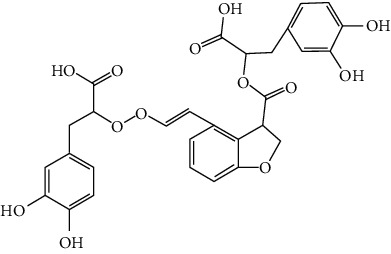	
Rosmarinic acid	Salvianolic acid A	Salvianolic acid B	

**Table 5:** Neuroprotective activity of other phenols and their mechanisms and targets^c^.

Compounds	Mechanisms and targets	Ref.
Other phenols (20)		
Creosol	Antiexcitotoxicity; Ca^2+^ influx↓	[243]
Curcumin	Antiautophagy: PI3K/Akt/mTOR↑; anti-inflammation: TLR4/p38/MAPK↓; antiapoptosis; GLUT1 and GLUT3↑; neurogenesis: Notch signaling pathway↑; antioxidation: Akt/Nrf2↑; mitochondrial protection: SIRT1↑; BBB protection	[10, 96, 244–249]
Cannabidiol	BBB protection; anti-inflammation; Na^+^/Ca^2+^ exchangers↑; antiapoptosis; antiexcitotoxicity; metabolic derangement↓	[250–252]
Hydroxytyrosol	Anti-inflammation; BDNF↑	[253]
Acteoside	Antioxidation; antiapoptosis	[254]
Hydroquinone	BBB protection: SMI-71↑, GLUT-1↑, ZO-1↓, occludin degradation↓	[255]
Lyciumamide A	Antioxidation: PKC*ε*/Nrf2/HO-1↑; antiapoptosis	[256]
Oleuropein	Antiapoptosis: Bcl-2/Bax↑, Akt↑/GSK3*β*↓	[257, 258]
Salidroside	Anti-inflammation: microglial M2 polarization↑, PI3K↑/PKB↑/Nrf2↑/NF-*κ*B↓, PI3K/Akt/HIF*α*↑; antiapoptosis: BDNF/PI3K/Akt↑; complement C3 activation↓; Egrs expression↑	[71, 259–264]
6-Shogaol	Anti-inflammation: CysLT1R↓, MAPK↓	[265]
4-Hydroxybenzyl alcohol	Antioxidation	[266]
4-Methoxy benzyl alcohol	BBB protection: NOS pathway↓, AQP-4↓, tight junction↑	[267]
Cinnamophilin	Gray and white matter damage↓	[268]
Hyperforin	TRPC6/MEK/Erk/CREB↑; TRPC6/CaMKIV/CREB↑	[269]
Punicalagin	Antioxidation; anti-inflammation; antiapoptosis	[270, 271]
Caffeic acid	Antioxidation; anti-inflammation: 5-LOX↓; loss of neuronal cells↓; synaptic density and plasticity↑	[80, 272]
Ferulic acid	Peroxiredoxin-2↑; thioredoxin↑	[273]
Gallic acid	Antiapoptosis and mitochondrial protection: Erk↑/cyclophilin D↓/mPTP↓	[99, 274]
Rosmarinic acid	Anti-inflammation: HMGB1/NF-*κ*B↓; synaptogenic activity↑; BDNF↑; BBB protection	[275, 276]
Salvianolic acid A	Antioxidation; anti-inflammation; metabolic dysfunction↓	[277]

^c^Notes: ↑: activation or upregulation; ↓: inhibition or downregulation. Abbreviations do not appear in the text. CysLT1R: cysteinyl leukotriene receptor 1; ZO-1: zonula occludens 1.

**Table 6:** Neuroprotective terpenoids and their functional mechanisms and targets^d^.

Compounds	Mechanisms and targets	Ref.
Monoterpenoids (14)		
Borneol	Antiapoptosis; anti-inflammation; neurovascular unit function↑	[289, 290]
Carvacrol	Ferroptosis↓; antioxidation; GPx4↑; TRPM7↓; antiapoptosis: Bcl-2/Bax↑, PI3K/Akt↑; anti-inflammation: NF-*κ*B↓	[46, 291–293]
Catalpol	Angiogenesis↑; JAK2/STAT3↑; ATPase activity↑; excitatory amino acid toxicity↓	[294, 295]
Cornin	Mitochondrial protection; antioxidation	[296]
Genipin	Antiapoptosis; UCP2/SIRT3↓	[297]
Geniposide	Antiapoptosis; BBB protection; GluN2A/Akt/Erk↑	[41]
Linalool	Phospholipid homeostasis↑	[298]
*β*-Myrcene	Antioxidation: free radical scavenging	[299]
Paeoniflorin	Calcium modulation: Ca^2+^↓/CaMKII↑/CREB↑; anti-inflammation: MAPK/NF-*κ*B↓; antiapoptosis	[300–302]
Perillaldehyde	Anti-inflammation: JNK↓; antiapoptosis: Akt↑	[303]
Perillyl alcohol	Anti-inflammation; antioxidation	[304]
*α*-Pinene	Antioxidation; anti-inflammation	[305]
Picroside II	Antioxidation: Rac-1/NOX2↓; antiapoptosis: mPTP permeability↓; anti-inflammation: MEK/Erk1/2/COX-2↓; BBB protection: ROCK/MLCK/MMP-2↓/claudin-5↑	[56, 79, 100, 306, 307]
Safranal	Antioxidation	[308]
Sesquiterpenoids (8)		
Alantolactone	Anti-inflammation: MAPK/NF-*κ*B↓	[312]
Atractylenolide III	Anti-inflammation: mitochondrial fission in microglia↓, JAK2/STAT3/Drp-1↓	[86]
Bakkenolide IIIa	Antioxidation; anti-inflammation: Erk↓, Akt/NF-*κ*B↓	[313]
Bilobalide	Mitochondrial protection: complex I function↑; antiexcitotoxicity; anti-inflammation: JNK1/2↓, p38 MAPK↓; antiautophagy; antiapoptosis; angiogenesis↑: Akt/eNOS↑	[314–317]
(−)-*α*-Bisabolol	Anti-inflammation	[318]
Parthenolide	BBB permeability↓; caspase-1/p38/NF-*κ*B↓	[319]
Patchouli alcohol	Anti-inflammation	[320]
*β*-Caryophyllene	Anti-inflammation: microglial M2 polarization↑, TLR4↓	[321]
Diterpenoids (11)		
Andrographolide	Anti-inflammation: microglial activation↓, PI3K/Akt-NF-*κ*B/HIF-1*α*↓, astrocyte activation↓, iNOS; BBB permeability↓; antioxidation: p38/Nrf2/HO-1↑, gp91^phox^/NOX2↓; BDNF/TrkB↑	[57, 325–328]
Erinacine A	Anti-inflammation: iNOS, p38, and CHOP↓	[329]
Ginkgolide B	Anti-inflammation: microglial M2 polarization↑, NF-*κ*B↓; PAF receptor↓; antiapoptosis; antiexcitotoxicity: imbalance of excitatory and inhibitory amino acids↓; BBB permeability↓	[38, 72, 330, 331]
Ginkgolide K	Antioxidation; neurogenesis: JAK2/STAT3↑	[11, 332]
Pseudopterosin A	Antioxidation; anti-inflammation; antiapoptosis: Akt↑	[333]
Salvinorin A	Mitochondrial function↑: AMPK/Mfn2↑, kappa opioid receptor↑	[334]
Tanshinone I	Neuronal death↓; anti-inflammation	[335]
Tanshinone IIA	Antiapoptosis: PI3K/Akt↑; anti-inflammation: HMGB1/NF-*κ*B↓, MIF/NF-*κ*B↓, astrocyte activation, MAPKs↓, PPAR*γ*↑; antioxidation; TORC1↑; BDNF/CREB↑	[75, 336–343]
Totarol	Antioxidation: Akt/HO-1↑	[344]
Triptolide	BBB permeability↓; anti-inflammation: p38/NF-*κ*B↓; autophagy↑; antiapoptosis	[113, 345, 346]
(1S,2E,4R,6R,-7E,11E)-2,7,11-Cembratriene-4,6-diol	Antiapoptosis: PI3K/Akt↑; ICAM-1↓	[347]
Triterpenoids (20)		
Arjunolic acid	Antioxidation	[357]
Asiatic acid	Antiapoptosis and mitochondrial protection: cytochrome c and AIF release↓; MMP-9↓	[358, 359]
Acetyl-11-keto-*β*-boswellic acid	Antioxidation: Nrf2/HO-1↑; anti-inflammation: 5-LOX, NF-*κ*B↓	[81, 360, 361]
11-Keto-*β*-boswellic acid	Antioxidation: Nrf2/HO-1↑	Ding et al. (2015)
28-O-Caffeoyl betulin	Anti-inflammation; hypothermic effects	[362]
Celastrol	Anti-inflammation: microglial M2 polarization↑, IL-33/ST2↓, JNK/c-Jun/NF-*κ*B↓	[73, 363]
Echinocystic acid	Antiapoptosis; anti-inflammation: JNK↓	[364]
18*β*-Glycyrrhetinic acid	Antioxidation; antiapoptosis	[365]
Maslinic acid	Synaptogenesis↑: axonal regeneration↑, Akt/GSK-3*β*↑	[366]
Ursolic acid	Anti-inflammation; antioxidation: Nrf2↑	[367]
Madecassoside	Antioxidation; antiapoptosis; anti-inflammation	[368]
Astragaloside IV	Antiapoptosis: P62-LC3-autophagy↑; antioxidation: Nrf2↑; mitochondrial protection: Akt/hexokinase-II↑; anti-inflammation	[62, 114, 369, 370]
Glycyrrhizin	Anti-inflammation: HMGB1/TLR4/IL-17A↓; antioxidation; antiexcitotoxicity; antiapoptosis	[91, 371–373]
Diammonium glycyrrhizinate	Anti-inflammation	[374]
Ginsenoside Rb1	BBB protection; anti-inflammation; antioxidation: NOX4-derived ROS production↓; abnormal microenvironment↓: glutamate toxicity↓, Ca^2+^ accumulation↓, GLT-1↑, NMDAR↓; autophagy↑; neurogenesis↑; BDNF↑; caspase-3↓	[34, 55, 115, 375, 376]
Ginsenoside Rd	Anti-inflammation: microglial proteasome-mediated NF-*κ*B activation↓, PARP-1↓; antioxidation: free radical scavenging; antiapoptosis; mitochondrial protection; energy restoration; Ca^2+^ modulation: TRPM7↓, ASIC1 a↓, ASIC2 a↑; DNA damage↓: NEIL1/3↑	[49, 377–383]
Ginsenoside Rg1	Anti-inflammation: microglial proteasome-mediated NF-*κ*B activation↓; BDNF↑; excitatory amino acid↓; antioxidation: miR-144↓/Nrf2↑/ARE↑; angiogenesis↑: PI3K/Akt/mTOR↑; BBB permeability↓: aquaporin-4↓, PAR-1↓	[384–389]
20(R)-Ginsenoside Rg3	Antiapoptosis: calpain I↓, caspase-3↓	[390]
Pseudoginsenoside F11	Antiapoptosis; autophagic/lysosomal defects↓; Ca^2+^ overload↓	[391, 392]
Notoginsenoside R1	Antiapoptosis; mitochondrial protection; estrogen receptor-Akt/Nrf2↑	[393]
Tetraterpenoids (3)		
Astaxanthin	Antioxidation; antiapoptosis; neurogenesis↑; neurotrophin expression: BDNF↑, NGF↑	[120, 400, 401]
Fucoxanthin	Antioxidation: Nrf2/HO-1↑	[402]
Lutein	Antiapoptosis; antioxidation; anti-inflammation	[403]

^d^Notes: ↑: activation or upregulation; ↓: inhibition or downregulation. Abbreviations do not appear in the text. CHOP: C/EBP homologous protein; GPx4: glutathione peroxidase 4; MLCK: myosin light chain kinase; PAR-1: protease-activated receptors; ROCK: Rho-associated kinase.

**Table 7:** Chemical structures of some representative neuroprotective terpenoids and alkaloids.

Monoterpenoids			
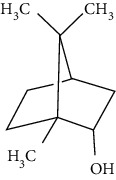	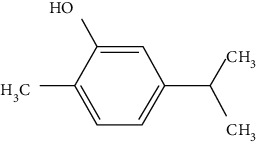	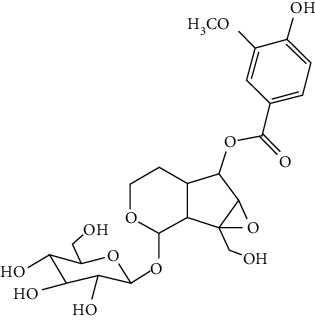	
(+)-Borneol	Carvacrol	Picroside II	
Sesquiterpenoids and diterpenoids			
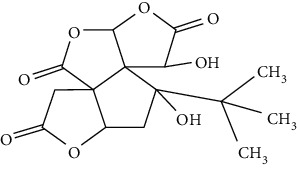	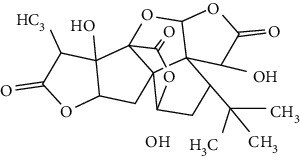	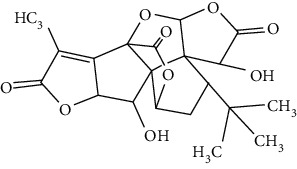	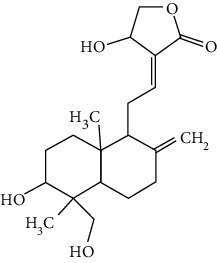
Bilobalide	Ginkgolide B	Ginkgolide K	Andrographolide
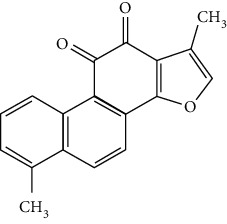	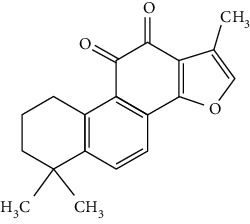	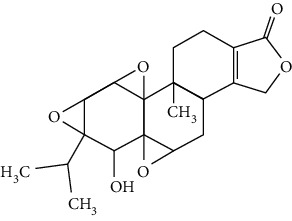	
Tanshinone I	Tanshinone IIA	Triptolide	
Triterpenoids and tetraterpenoids			
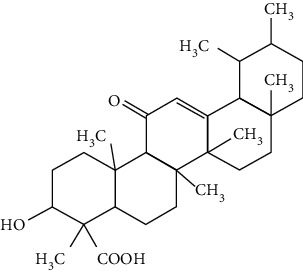	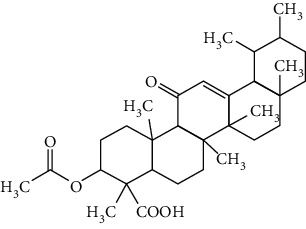	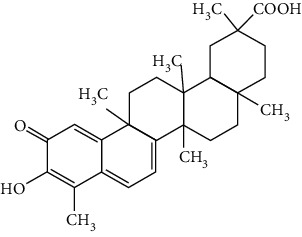	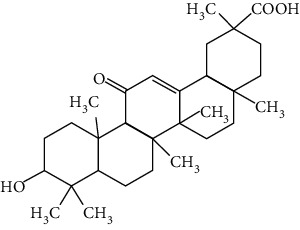
11-Keto-*β*-boswellic acid (KBA)	Acetyl-11-keto-*β*-boswellic acid (AKBA)	Celastrol	Glycyrrhetinic acid
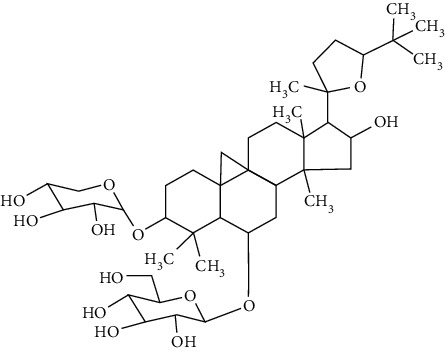		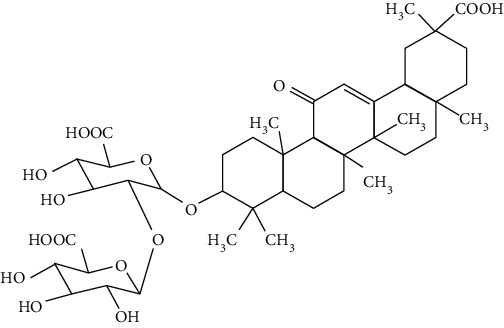	
Astragaloside IV		Glycyrrhizin	
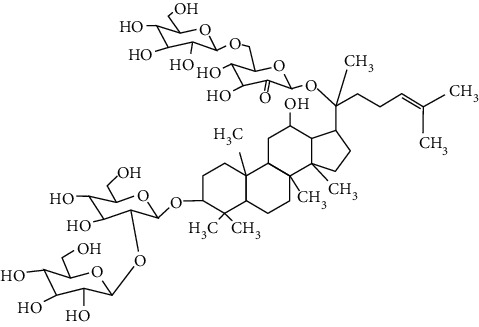		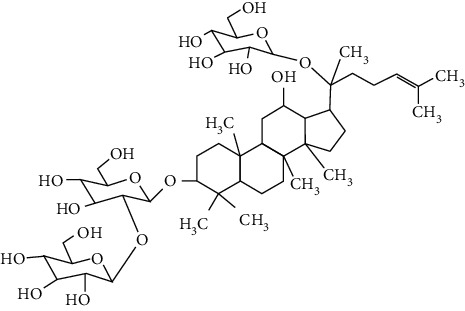	
Ginsenoside Rb1		Ginsenoside Rd	
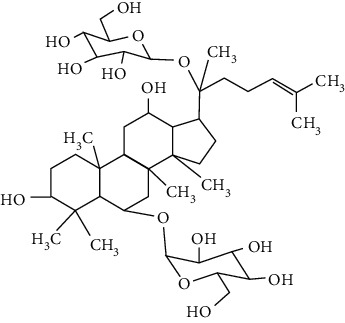		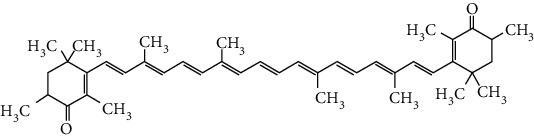	
Ginsenoside Rg1		Astaxanthin	
Alkaloids			
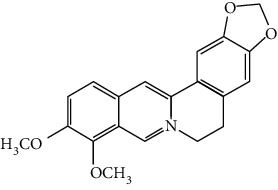	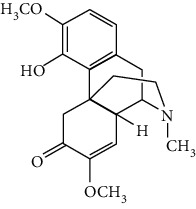	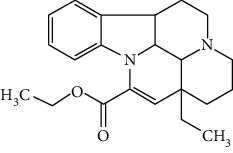	
Berberine	Sinomenine	Vinpocetine	
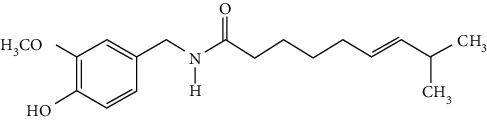		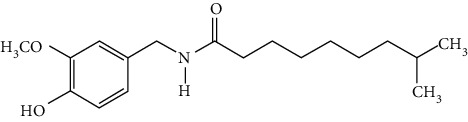	
Capsaicin		Dihydrocapsaicin	

**Table 8:** Neuroprotective alkaloids and their functional mechanisms and targets^e^.

Compounds	Mechanisms and targets	Ref.
Alkaloids (19)		
Berberine	Antiapoptosis: BDNF-TrkB-PI3K/Akt↑, PI3K p55*γ* activity↑, Akt/GSK↑; angiogenesis↑; claudin-5↑; anti-inflammation: microglial and astrocyte activation↓, AMPK-dependent microglial M2 polarization↑, HMGB1/TLR4/NF-*κ*B↓	[74, 92, 405–409]
Boldine	Anti-inflammation	[410]
Capsaicin	Antiexcitotoxicity: TRPV1-dependent inhibition of NMDA receptors↑; neurovascular protection	[47, 411]
Dihydrocapsaicin	Hypothermia: TRPV1↓; PI3K/Akt↑; BBB protection; antioxidation; anti-inflammation	[44, 412–414]
Harmine	GLT-1↑; astrocyte activation↓	[35]
Higenamine	HMGB1↓; PI3K/Akt/Nrf2/HO-1↑	[415]
Neferine	Mitochondrial protection: Nrf2 pathway↑	[63]
Nicotine	Anti-inflammation: microglial proliferation↓, *α*7nAchR↑	[416]
Levo-tetrahydropalmatine	Antiapoptosis: c-Abl↓	[417]
Oxymatrine	Anti-inflammation: arachidonic acid release↓, 12/15-LOX/p38 MAPK/cPLA2↓; Nrf2/HO-1↑	[83, 418]
Oxysophoridine	Antiapoptosis	[419]
Sophoridine	Antiapoptosis; ASIC1↓; TRAF6↓/Erk1/2↑	[425, 426]
Piperine	Anti-inflammation	[420]
Rhynchophylline	PI3K/Akt/mTOR↑	[421]
Sinomenine	Anti-inflammation: NLRP3 inflammasomes↓, DRD2↑/CRYAB↑/STAT3↓; AMPK↑; acidosis↓: ASIC1a↓	[87, 422, 423]
Solasodine	Antioxidation	[424]
Tetrandrine	GRP78 and HYOU1↓; DJ-1↑	[427]
Trigonelline	Glutathione-mediated myeloperoxidase expression↓	[428]
Vinpocetine	Anti-inflammation: TLR4/MyD88/NF-*κ*B↓	[93, 429]

^e^Notes: ↑: activation or upregulation; ↓: inhibition or downregulation. Abbreviations do not appear in the text. c-Abl: nonreceptor Abelson tyrosine kinase; DJ-1: PARK7, Parkinsonism associated deglycase; GRP78: glucose-regulated protein of 78 kDa; HYOU1: hypoxia upregulated protein 1.
